# Genome Analysis of *Bacillus amyloliquefaciens* Subsp. *plantarum* UCMB5113: A Rhizobacterium That Improves Plant Growth and Stress Management

**DOI:** 10.1371/journal.pone.0104651

**Published:** 2014-08-13

**Authors:** Adnan Niazi, Shahid Manzoor, Shashidar Asari, Sarosh Bejai, Johan Meijer, Erik Bongcam-Rudloff

**Affiliations:** 1 Department of Animal Breeding and Genetics, SLU Global Bioinformatics Centre, Swedish University of Agricultural Sciences, Uppsala, Sweden; 2 Department of Plant Biology, Linnéan Center for Plant Biology, Uppsala Biocenter, Swedish University of Agricultural Sciences, Uppsala, Sweden; 3 University of the Punjab, Lahore, Pakistan; Agriculture and Agri-Food Canada, Canada

## Abstract

The *Bacillus amyloliquefaciens* subsp. *plantarum* strain UCMB5113 is a Gram-positive rhizobacterium that can colonize plant roots and stimulate plant growth and defense based on unknown mechanisms. This reinforcement of plants may provide protection to various forms of biotic and abiotic stress. To determine the genetic traits involved in the mechanism of plant-bacteria association, the genome sequence of UCMB5113 was obtained by assembling paired-end Illumina reads. The assembled chromosome of 3,889,532 bp was predicted to encode 3,656 proteins. Genes that potentially contribute to plant growth promotion such as indole-3-acetic acid (IAA) biosynthesis, acetoin synthesis and siderophore production were identified. Moreover, annotation identified putative genes responsible for non-ribosomal synthesis of secondary metabolites and genes supporting environment fitness of UCMB5113 including drug and metal resistance. A large number of genes encoding a diverse set of secretory proteins, enzymes of primary and secondary metabolism and carbohydrate active enzymes were found which reflect a high capacity to degrade various rhizosphere macromolecules. Additionally, many predicted membrane transporters provides the bacterium with efficient uptake capabilities of several nutrients. Although, UCMB5113 has the possibility to produce antibiotics and biosurfactants, the protective effect of plants to pathogens seems to be indirect and due to priming of plant induced systemic resistance. The availability of the genome enables identification of genes and their function underpinning beneficial interactions of UCMB5113 with plants.

## Introduction

Plant growth-promoting rhizobacteria (PGPR) colonize the plant rhizosphere and develop close physical and biochemical contacts with plants and enhance growth of the plant [1]. Many PGPR can also help plants to tolerate abiotic and or biotic stress better [2], [3]. PGPR can mediate plant growth and protection in several ways. For instance, competition for growth space and essential nutrients, and production of a wide range of antibiotics and enzymes (like proteases and chitinases) counteract harmful microorganisms. Moreover production of siderophores also protect plants by solubilizing and scavenging iron from the environment, hence making it unavailable for other and more deleterious microorganisms [4]. Phytohormones such as auxins, gibberellins, and cytokinins produced by certain bacteria stimulate growth of the plants [5].

Many *Bacillus* ssp. have been found to provide beneficial effects to different plant species [6], [7]. The Gram-positive *Bacillus* group represents a large genetic and habitat diversity and has several properties of interest for applied use. Many *Bacillus* that can serve as biofertilizers and biopesticides are regarded non-pathogenic which makes handling easier. These bacteria are mobile and show good rhizosphere competence and are also facultative anaerobes enabling survival in soil at different environmental conditions. *Bacillus* sporulate under unfavorable conditions and the spores are very resistant to harsh conditions providing long shelf-life useful for commercial applications. Several *Bacillus* ssp. have high secretory capacity and certain strains are used as “cell factories" for industrial production of enzymes. *Bacillus subtilis* is one of the best studied bacteria out of many aspects impoving understanding of many processes and features. *B. amyloliquefaciens* subsp. *plantarum* strains are capable to enhance plant growth and confer protection by producing phytohormones and antimicrobial compounds. The type strain of the *B. amyloliquefaciens* subsp. *plantarum* group, FZB42, is known to have a great capacity for non-ribosomal synthesis of secondary metabolites including lipopeptides and polyketides by some gene clusters with antimicrobial and antifungal activity [8], [9].

The plant root-colonizing strain *B. amyloliquefaciens* subsp. *plantarum* UCMB5113 was isolated from soil in the Karpaty mountains of Ukraine and was identified initially as a member of the *B. amyloliquefaciens* group on the basis of phenotypic properties and partial gene sequence analysis [10]. The UCMB5113 strain can promote growth of both underground and aboveground tissues of different plants. This is observed as increased root branching, increased total root surface area, and increased leaf area providing increased photosynthesis capacity, and nutrient and water uptake. The UCMB5113 strain can restrict the growth of several fungal pathogens on oilseed rape such as *Alternaria brassicae*, *Botrytis cinerea*, *Leptosphaeria maculans* and *Verticillium longisporum* [11], [12]. Disease suppression by UCMB5113 has also been observed using *Arabidopsis thaliana* infected with fungal or bacterial pathogens (unpublished observations). Further, improved tolerance of UCMB5113 treated wheat seedlings to abiotic stress factors like drought, cold and heat has been demonstrated [13], [14], [15]. Thus, the UCMB5113 strain seems to have the capacity to operate on different plants, improve different kinds of stress management and stimulate plant growth making it an interesting candidate for use in agriculture to support more sustainable crop production.

In this study, we describe the analysis of the *B. amyloliquefaciens* subsp. *plantarum* UCMB5113 genome sequence, and through comparison with the model species of the *B. subtilis* group, make an attempt to target genes that contribute to the beneficial interaction between bacteria and plants that ultimately results in growth promotion and improved stress management of the host plant.

## Materials and Methods

### Genome sequencing and assembly

The genomic DNA isolated from *B. amyloliquefaciens* subsp. *plantarum* UCMB5113 was sequenced through multiplexed sequencing process using Illumina technology and read data from two sequencing lanes were used. A total of 16,399,248 paired-end reads of length 75 bp with the average insert size of 230 bp were generated. MIRA ver_3.4 [16] and Velvet ver_1.1.04 [17] was used for the assembly of reads-data with the similar assembly approach as previously applied [18]. The published genome of *B. amyloliquefaciens* FZB42 (*accession no*. NC_009725) [19] was used as template to identify genomic rearrangements using Mauve genome alignment software [20]. All gap-filled regions and indels were verified through genomic PCR and subsequent Sanger sequencing of the amplicons.

### Genome annotation and comparative analysis

The genome annotation was accomplished via the Magnifying Genome (MaGe) Annotation Platform [21] which is embedded with tools for structural annotation and functional annotation. Putative functions to encoding genes were automatically assigned via BlastP similiarity searches against the UniProt database. PRIAM was used to predict enzyme-coding genes, and transmemberane domains were identified by THMM. All of these tools are stitched up in MaGe annotation pipeline. Lastly, the predictions were reviewed and curated manually.

Comparative analysis of the genome was performed using the EDGAR software framework for the prokaryotic genomes [22] to identify homologs and non-homologs in all of the selected genomes of closely related *Bacillus* species. For phylogenetic analysis, complete sequences of nine housekeeping genes from *B. amyloliquefaciens* and other *Bacillus* species were obtained and concatenated after performing multiple alignment using MUSCLE [23]. Phylogenetic tree was constructed by the Maximum-Likelihood method with bootstrap analysis (1000 replications) using MEGA5 [24]. The genetic content comparison was conducted at protein level using BLASTP program (min length ≥80 and identity ≥80) between the completely available genomes of ‘*plantarum*’ and ‘*amyloliquefaciens*’ species. Similarity between complete genomes and local genome regions were visualized with GenoPlotR.

Regions of genome plasticity (RGP) were identified using RGP finder, SIGI, and IVOM programs embedded in the MAGE platform, by taking into account some genomic properties which include synteny breakage, sequence composition (GC-content and codon bias), tRNA, phage, and mobile elements spots such as recombinases, IS elements, integrases and transposases. ISFinder (http://www.is.biotoul.fr/) was used for the identification of IS elements. The prediction of secretory proteins containing signal peptides was done using SignalP v4.0 [25], lipoproteins containing cleavage sites for signal peptidase II (SPII) were predicted using LipoP v1.0 [26], and proteins with Twin-arginine cleavage sites were identified by TatP1.0 [27].

The complete nucleotide genome sequence of *B. amyloliquefaciens* subsp. *plantarum* UCMB5113 has been deposited in European Nucleotide Archive (ENA) database under accession number HG328254.

### Carbohydrate fermentation analysis

Fermentation analysis of several sugars was performed using the API 50 CH system (BioMerieux). The fermentation test strips were inoculated with UCMB5113 resuspended in API 50 CHB/E Medium and was incubated for 24–48 hours at 28°C under aerobic conditions.

### Enzyme activity assays

A single bacterial colony was streaked and/or 50 µl of 1×10^7^ CFU ml^−1^ bacterial culture was inoculated at the center of agar plates containing different carbon source media and incubated at 28°C for 2–4 days. The assays were conducted as described using blood agar assay [28]; drop collapse test [29]; siderophore assay [30]; chitinase assay – crab-shell chitin [31] dissolved in M9 minimal medium without glucose; phosphate solubilization assay [32]; starch hydrolysis [33] and urease activity [34]. All the experiments were repeated at least twice with similar results and a representative experimental result is shown.

### Swarming motility

In three independent experiments, aliquots of 50 µl of 1×10^7^ bacterial culture were inoculated at the center of PDA (Potato dextrose agar) plates and incubated at 28°C for 4 days.

### UCMB5113 mediated growth promotion of *Brassica napus* and *Arabidopsis thaliana*

*Brassica napus* cv. Westar and *Arabidopsis thaliana* ecotype Col-0 seeds were surface sterilized (1 min in 70% ethanol, 5 min in 10% bleach containing chlorine and Tween 20, and 5 min in water repeated 3 times). The seeds were dipped in a spore suspension of 1×10^7^ CFU/ml^−1^ of *Bacillus* UCMB5113 and incubated at 28°C for 2 h. The seeds were then sown on sterile soil (s-soil, Hasselfors Garden), one seed per pot and grown at 22°C, 16/8 h photo period, for 18 to 21 days.

*In vitro* growth promotion studies were carried out using *Arabidopsis* seeds sterilized as above but germinated on Murashige & Skoog (MS) nutrient medium containing 0.6% bacto agar for 12 days at 22°C, 16/8 h photo period. Later the seedlings were dipped in a solution with 1×10^7^ CFU ml^−1^ *Bacillus* strain UCMB 5113 and transplanted on 22×22 cm square plates containing MS with 0.8% bacto agar. The plants were grown at 22°C, 16/8 h photo period for two weeks further and growth promotion parameters were recorded.

## Results and Discussion

### Genomic structure and features of *B*. *amyloliquefaciens* UCMB5113

**Figure 1:**
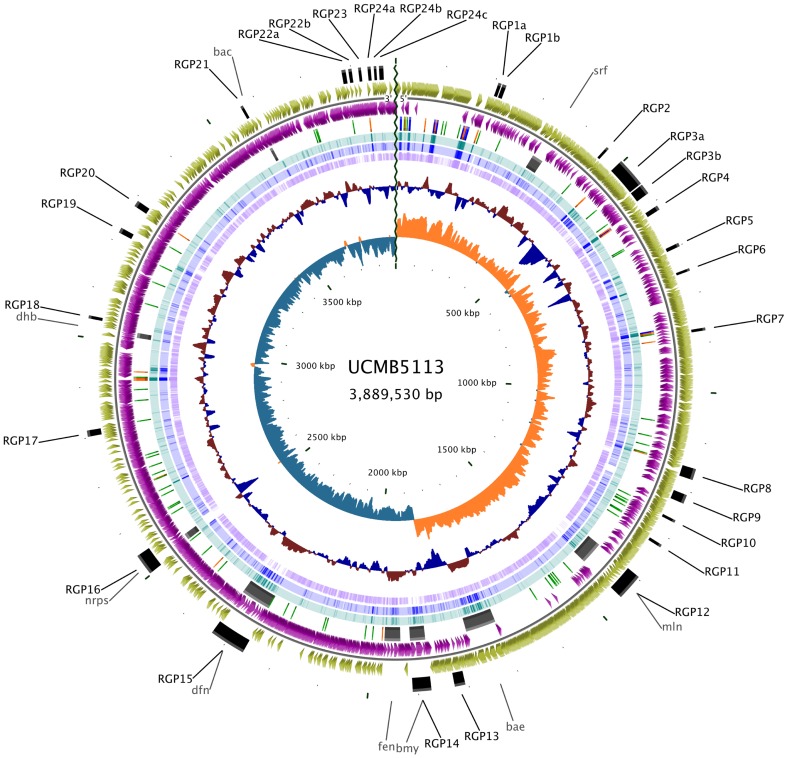
Graphical representation of genomic features of *B. amyloliquefaciens* subsp. *plantarum* UCMB5113. Circles display (from the outside): (1) Sites of genome plasticity. (2) Predicted CDSs transcribed in the clockwise direction. (3) Predicted CDSs transcribed in the counterclockwise direction. (4) rRNA (blue), tRNA (orange), non-coding RNA (green), and NRPS/PKS gene clusters (grey). (5,6,7) Blast comparison of UCMB5113 genome with type strain FZB42^T^ and *B. subtilis* 168, respectively. (8) GC percent deviation (GC window - mean GC) in a 1000-bp window. (9) GC skew (G+C/G-C) in a 1000-bp window.

The general genomic structure of the *B. amyloliquefaciens* subsp. *plantarum* UCMB5113 chromosome of 3,889,530 bp is depicted in Figure 1. The genome was predicted to encode 3,656 coding sequences (CDSs) including 4 fragmented coding sequences (fCDSs). Putative biological functions were assigned to 3,106 CDS (85%) after manual curation. There were 506 (13.7%) conserved hypothetical proteins, and 44 (1.2%) hypothetical proteins having no homology to any previously reported sequence. The genome contains 10 copies of 5S, 16S, and 23S rRNA genes. Analysis of tRNAs showed 89 tRNA genes with specificities for all 20 standard amino acids on the chromosome.

**Figure 2:**
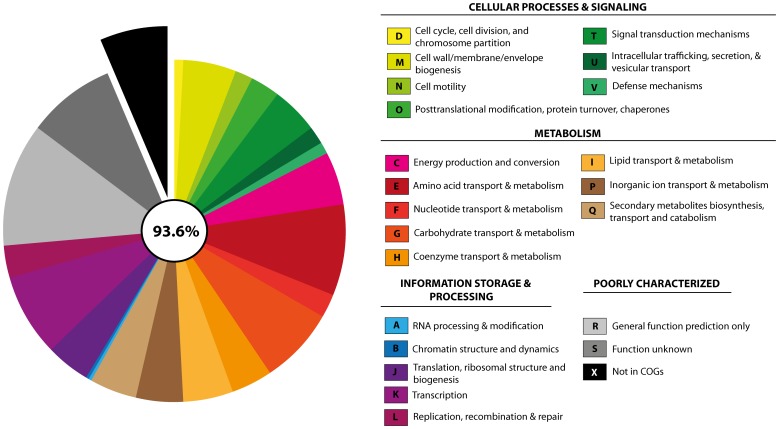
Functional classification of protein-coding genes in UCMB5113. Distibution of UCMB5113 coding sequences (93.6%) in COG functional classes. Genes that did not have any inferred COG annotation were assigned to category X.

The functional classification assigned 3,437 CDS in different COG (cluster of orthologs groups) functional groups (Figure 2). This analysis revealed that, apart from essential housekeeping genes, the genome seems biased towards three major functional gene classes: amino acid (E), carbohydrate (G), inorganic ion (P) transport and metabolism, secondary metabolite biosynthesis (Q), and defense mechanisms (V) representing 26% of all CDS. The high proportion of these genes in the genome indicates the inherent potential of UCMB5113 to compete in the rhizosphere with other microorganisms by having an efficient uptake of nutrients, along with the set of genes needed for defense mechanisms and secondary metabolite biosynthesis to antagonize other microorganisms.

### Comparative analysis and phylogeny

**Figure 3:**
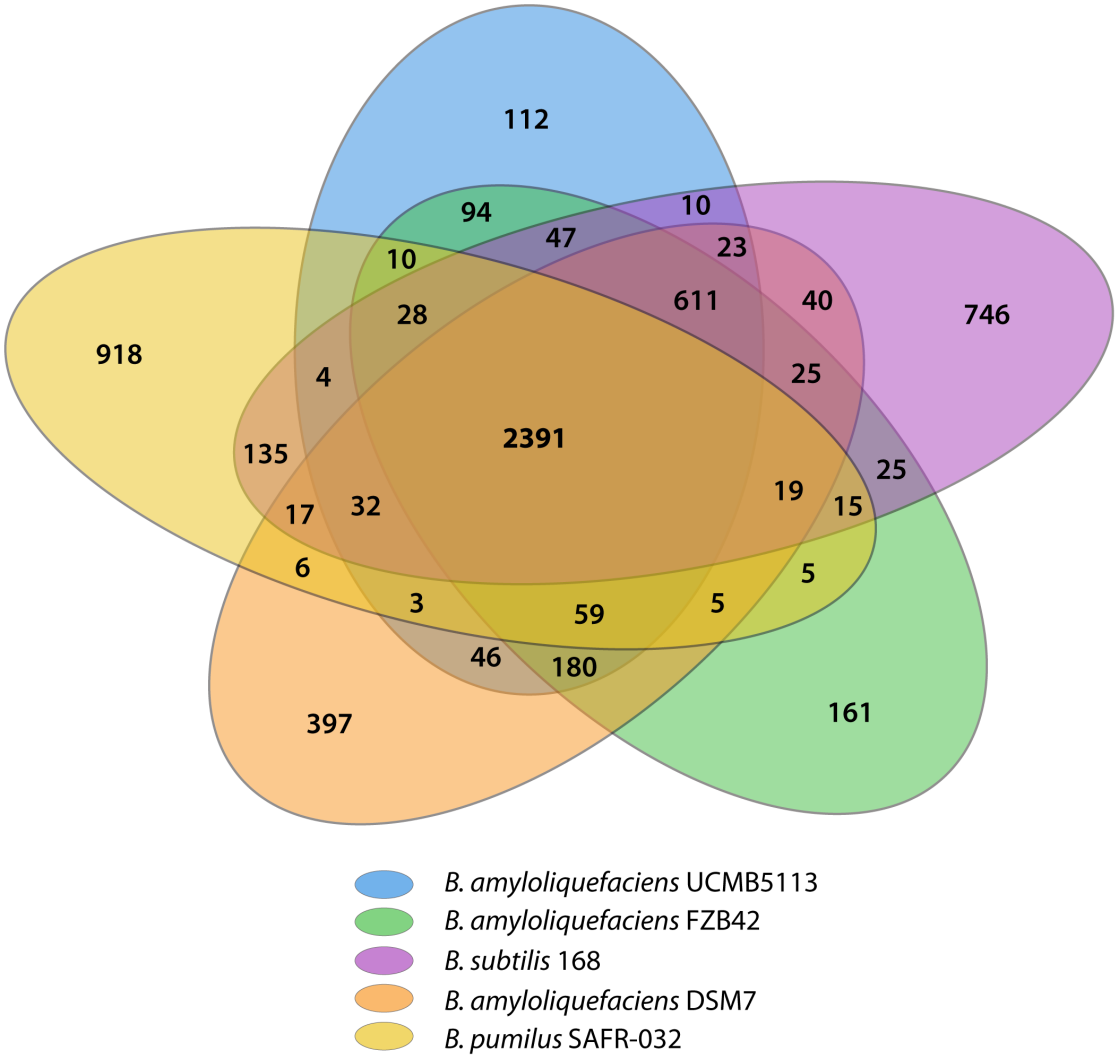
Numbers of shared and genome-specific genes. The Venn Diagram shows the number of shared and genome-specific genes in *B. amyloliquefaciens* subsp. *plantarum* UCMB5113, *B. amyloliquefaciens* subsp. *plantarum* FZB42^T^, *B. subtilis* 168, *B. amyloliquefaciens* subsp. *amyloliquefaciens* DSM7^T^ and *B. pumilus* SAFR-032.

**Figure 4:**
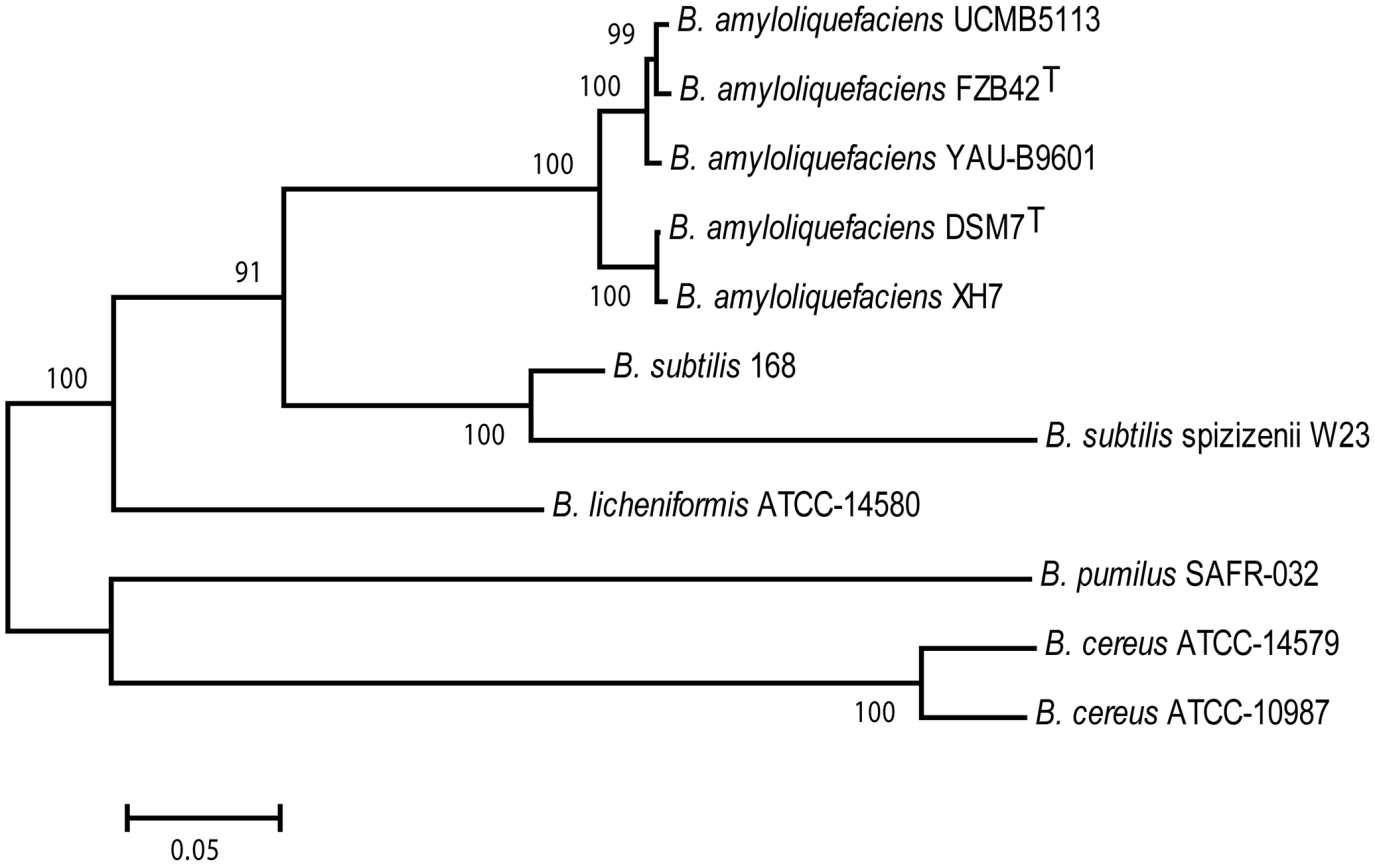
Neighbour-joining phylogenetic tree. The position of *B. amyloliquefaciens* strain UCMB5113 in relation to other species within the genus *Bacillus*. The numbers above the branches are support values obtained from 1,000 bootstrap replicates.

A comparative analysis of the *B. amyloliquefaciens* subsp. *plantarum* UCMB5113 genome with other *Bacillus* species is shown in Table 1. The over all core genome of these species is comprised of 2391 orthologs (Figure 3). In total, 3,077 coding sequences are shared by the genomes of *B. subtilis* 168, *B. amyloliquefaciens* FZB42 and *B. amyloliquefaciens* UCMB5113. The genomes of UCMB5113 and FZB42 appear to be most similar by sharing 3,421 orthologs, whereas 3,345 orthologs were found between UCMB5113 and the non plant-associated type strain *B. amyloliquefaciens* subsp. *amyloliquefaciens* DSM7^T^. A total of 112 unique coding sequences in the genome of *B. amyloliquefaciens* subsp. *plantarum* UCMB5113 were identified in the comparison. Moreover, the seventh allele of the 16S rRNA gene was also found to be truncated similar to FZB42 indicating a close relationship and may explain the evolutionary aspects of the two strains. Phylogenetic analysis performed (Figure 4) using sequences of nine housekeeping genes (*gyrA*, *dnaX, glyA, cysS, glpF, gmk, gyrB, ligA* and *recN*) from *B. amyloliquefaciens* strains and other *Bacillus* species further illustrated the close relationship between these organisms. The tree also show the clear distinction that can be made between the subsp. *plantarum* and subsp. *amyloliquefaciens* [35].

**Table 1:** Comparison of genomic features of *B. amyloliquefaciens* subsp. *plantarum* UCMB5113 with genomes of other *Bacillus* spp. belonging to the *B. subtilis* group.

Features	*B. amyloliquefaciens*				
	UCMB5113	FZB42^T^	DSM7^T^	*B. subtilis* 168^T^	*B. pumilus* SAFR-032
Genome size (bp)	3,889,530	3,918,591	3,980,199	4,214,630	3,704,465
G+C content (%)	46.71	46.49	46.1	43.51	41.3
Protein-coding sequences	3656	3693	3893	4106	3679
Number of CDS shared	n.a*	3421	3345	3153	2541
Unique CDS (not shared by UCMB5113)	n.a	235	311	503	1115
rRNA operons	10	10	10	10	7
tRNA genes	89	89	94	86	69

*n.a: not applicable.

The genome of *B. amyloliquefaciens* subsp. *plantarum* UCMB5113 encodes several transcription regulators e.g. 17 sigma factors, 13 anti-sigma factors, and 2 anti-anti-sigma factors. The sigma factor encoding genes: *sigM, sigV, sigW, and sigX* were found while the genome is devoid of *sigY*, *sigZ* and *ylaC* and their antagonists *yxlC*, *sdss*, and *ylaD*, respectively. In addition, a putative sigma factor encoding gene *BASU_0627* was found similar to *sigV* (33% amino acid sequence identity).

### Regions of genome plasticity

The multiple genome alignment highlighted that the genome of *B. amyloliquefaciens* subsp. *plantarum* UCMB5113 shared large blocks within which the gene order and synteny appears to be remarkably conserved between the closely related organisms analyzed (Figure S1). However, UCMB5113 contains defined inserted regions that seem to have originated with the transfer of mobile genetic elements, including phages, transposases and insertion sequences. Several regions of genome plasticity where horizontal gene transfer often occurs were identified using RGP Finder. In total 29 sites of genome plasticity were identified by comparing the UCMB5113 genome with the available genome sequences of *B. subtilis* 168, *B. amyloliquefaciens* DSM7 and *B. amyloliquefaciens* FZB42. These regions carry genes involved in sporulation, competence development, metal and drug resistance, biosynthesis of metabolites, phage derived proteins, sugar transport (PTS system) as well as other transporters and regulators. Three of those regions (RGP3a, RGP16, RGP22) containing: nitroreductases, oxidoreductases, hydrolases, and putative NRPS related genes were specific to UCMB5113 (Figure 1, Table S1 in File S1), whereas one region corresponding to *B. subtilis* prophage elements was identified. Indels identified in the UCMB5113 genome are summarized in Table S2 in File S1.

### Prophages and transposable elements

The *B. amyloliquefaciens* subsp. *plantarum* UCMB5113 genome comprises 53 phage related genes including remnants and two putative phage integrases. A cluster of 16 phage-related genes (*xkdEFGHIJKMNOPQRSTU*) similar to *B. subtilis* was found on the chromosome. A significant portion of prophage derived endonuclease *yokF* was found truncated in the genome. Disruption of *yokF* in *B. subtilis* showed sensitivity to mitomycin C but increased transformation efficiency [36]. Two putative transposase encoding genes, *orfA* and *orfB*, identified with IS Finder (http://www-is.biotoul.fr/), as part of the only insertion sequence element, “ISBsu1” having a size of 1,289 bp from the IS3 family, exist in the UCMB5113 genome. Similar transposase genes seem conserved in most species of the *B. subtilis* group. Furthermore, three putative integrase (*BASU_1646, BASU_1806, BASU_1970*) and two resolvase encoding genes (*recU*, *yrrK*) involved in DNA recombination and decreasing susceptibility to DNA damaging agents were found on the chromosome.

### Extracellular enzymes and metabolic activities

**Figure 5:**
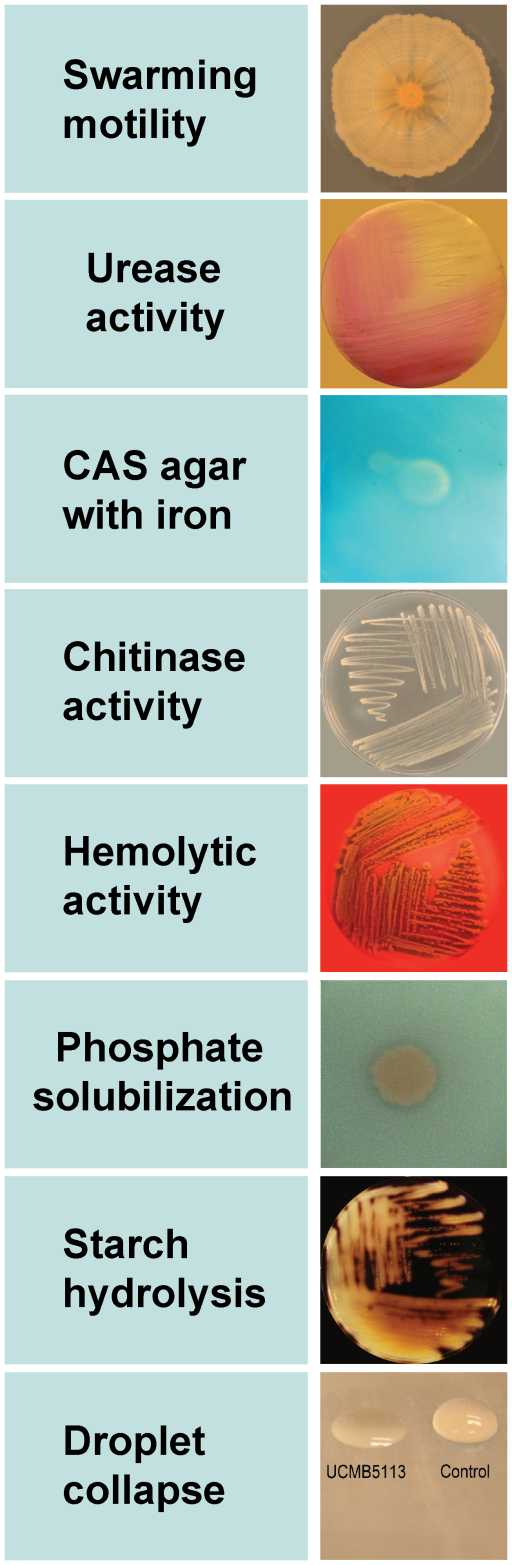
*B. amyloliquefaciens* UCMB5113 related activity on different substrates. A branch pattern with massive groups of bacteria observed after 4 days of incubation period indicated swarming motility; bright pink color indicated the hydrolysis of urea to carbon dioxide and ammonia; siderophore biosynthetic cluster produced a clear zone on CAS agar; chitin degradation and utilization as carbon source; expressed hemolytic activity on blood agar; phosphate solubilization around bacteria apparent as a transparent zone; amylase activity on starch medium; aqueous drop collapse to assess production of biosurfactants.

In the *B. amyloliquefaciens* subsp. *plantarum* UCMB5113 genome, 298 out of the 3,693 genes were predicted to encode proteins in the secretory pathway including 149 proteins that lack any transmembrane domain as predicted by THMM2 (Table S3 in File S1). Of these, 200 were Sec dependent having signal peptidase I as a site for cleavage, 87 were identified as lipoprotein consisting (SPII/Lsp), and 11 contained a TAT (twin-arginine translocation) motif for protein secretion. Out of all these, 78 were assigned putative functions, 26 were uncharacterized protein encoding genes, and 14 were hypothetical or conserved hypothetical genes. Of the remaining 180 genes, 111 were found to be enzyme encoding genes. Further examination revealed 11 unique putative CDSs in the genome predicted to be secreted in the extracellular space. In accordance with its symbiotic lifestyle, the secretome of UCMB5113 comprises various enzymes that probably hydrolyze polysaccharides, proteins and other compounds available in the rhizosphere. Part of this enzyme repertoire may be used by UCMB5113 to process plant surfaces to allow colonization at the rhizoplane. For instance, members of different glycoside hydrolase families (GH5, GH9, GH11, GH16, GH44, and GH48) may allow bacteria to grow on a broad range of polysaccharides by degrading hemicellulosic components present in plant cell walls. However, no putative members of the GH9, GH44, GH48 families were found in the UCMB5113 genome. Further degradative genes in UCMB5113 includes: *abnA* encoding endo-arabinase (GH43), *xynA* encoding xylanase (GH11), *bglS* encoding lichenase (GH16) and *eglS* encoding endo-glucanase/cellulase (GH5). Also members of the polysaccharide lyase family were identified which include pectate lyase (pel and BASU_3156) and pectin lyase (pelB) that may also be involved in degradation of plant tissues. All of the gene products encoded for polysaccharide degradation were predicted to have secretory signal peptides allowing degradation and assimilation of molecules present in the rhizosphere or rhizoplane. Tests to ensure the presence of certain prominent genes encoding hydrolytic enzymes revealed that UCMB5113 was able to utilize chitin, urea and starch (Figure 5).

### Secondary metabolites, siderophores and antibiotics

**Figure 6:**
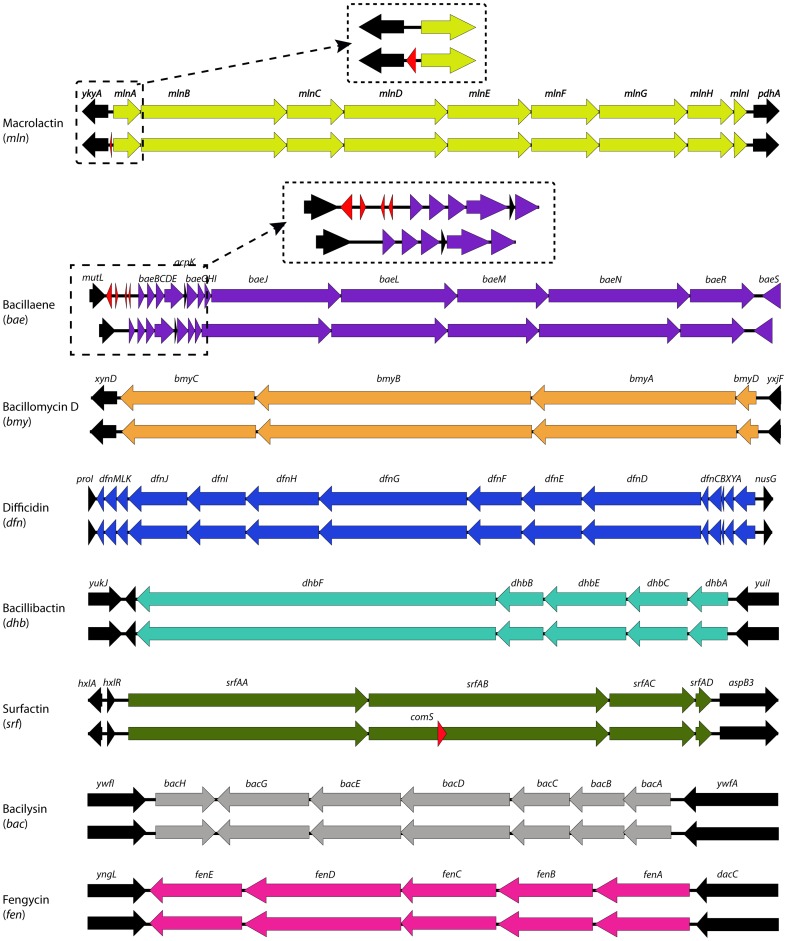
Blast comparison of NRPS/PKS clusters in UCMB5113 (above) and FZB42^T^(below). Arrows indicate gene clusters; Macrolactin (light green), Bacilllaene (purple), BacillomycinD (orange), Difficidin (blue), Bacillibactin (turquoise), Surfactin (green), Bacilysin (grey), Fengycin (pink). Genes highlighted in red represent the differences where as black represent other genes flanking in each cluster.

The genome of *B. amyloliquefaciens* subsp. *plantarum* UCMB5113 harbors gene clusters which are responsible for biosynthesis of several bioactive lipopeptides via nonribosomal peptide synthetases (NRPSs) including; surfactin (*srf*), bacillomycin D (*bmy*) and fengycin (*fen*), with known antagonistic activities (Table 2). A gene cluster for the dipeptide antibiotic bacilysin [37], [38], synthesized in several strains of *B. subtilis*, *B. amyloliquefaciens*, *B. pumilus*, and *B. licheniformis* [39], was also found in the UCMB5113 genome. In addition to NRPS operons, a macrolactin synthesizing *mln* operon that has been reported to exhibit antibacterial activity in *B. subtilis* AT29 [40] and *B. amyloliquefaciens* CHO104 [41] was located in the UCMB5113 genome. Furthermore, gene clusters for synthesis of bacillaene (*bae*) and difficidin (*dfn*) were identified in the chromosome. Both *bae* and *dfn* have been characterized to exhibit antimicrobial activity in *B. subtilis* and *B. amyloliquefaciens* strains [9], [42], [43]. The organization of gene clusters in UCMB5113 was observed to be similar to the corresponding genomic segments in FZB42 (Figure 6) with high identity at the amino acid level. Remnants of the *fen* gene cluster was located in the genome of *B. amyloliquefaciens* DSM7, whereas no counterparts for *mln* and *dfn* operons was found in the chromosomes of DSM7, *B. subtilis* 168, or sequenced species of *B. licheniformis, B. cereus*, and *B. pumilus*. In addition, a putative NRPS cluster of five genes (*BASU_2336-BASU_2340*) probably encoding a novel antibiotic was found on the chromosome of *B. amyloliquefaciens* subsp. *plantarum* UCMB5113. Apparently the UCMB5113 strain has great capacity for antibiosis and an earlier investigation showed that this strain could antagonize several phytopathogens using *in vitro* assays [11].

**Table 2:** NRPS and PKS gene clusters involved in synthesis of secondary metabolites in *B. amyloliquefaciens*.

Compound	UCMB5113	FZB42	DSM7^T^	Identity %
Surfactin	*srfABCD*	*srfABCD*	*srfABCD*	98–99
BacillomycinD	*bmyCBAD*	*bmyCBAD*	Not present	96–98
Fengycin	*fenABCDE*	*fenABCDE*	*fenDE*	98–99
Putative peptide	Not present	*nrsABCDEF*	Not present	0
Bacillibactin	*dhbACEBF*	*dhbACEBF*	*dhbACEBF*	95–99
Bacilysin	*bacABCDEGH*	*bacABCDEGH*	*bacABCDEGH*	99–100
Macrolactin	*mlnABCDEFGHI*	*mlnABCDEFGHI*	Not present	98–99
Bacillaene	*baeBCDE, acpk, baeGHIJLMNRS*	*baeBCDE, acpk, baeGHIJLMNRS*	*baeBCDE, acpk, baeGHIJLMNRS*	97–99
Difficidin	*dfnAYXBCDEFGHIJKLM*	*dfnAYXBCDEFGHIJKLM*	Not present	98–99
Putative peptide	*nrpsGFEC*	Not present	*nrpsGFEC*	0

Certain bacteria have developed diverse iron uptake mechanisms to compete for iron ions in the rhizosphere that often is a limiting factor for growth. Such mechanisms include iron uptake transporters, synthesis of siderophores and siderophore receptors, which contribute to confer protection of host plants from pathogens. The *B. amyloliquefaciens* subsp. *plantarum* UCMB5113 genome contains a gene cluster (*dhbABCDEF*) that is responsible for the synthesis of the iron-siderophore bacillibactin in *B. subtilis* [44]. The respective genes show identities of 95–99% for the *dhb* operon at the amino acid level. UCMB5113 grown on blood agar plates showed hemolytic activity within two days by the presence of a clear zone around the bacteria (Figure 5) indicating production of biosurfactants that can serve in antibiosis. Unlike *B. amyloliquefaciens* FZB42, no counterpart for the *nrs* operon, probably responsible for non-ribosomal synthesis of siderophores [17], was found in the genome of UCMB5113.

### Sporulation and competence genes

Some bacteria undergo morphological changes, i.e. sporulation, induced by nutritional depletion in the environment. Similar to *B. subtilis*, the chromosome of UCMB5113 possesses genes implicated in sporulation, including the *spsABCDEFG* operon responsible for the synthesis of spore coat polysaccharides. The exception, in contrast to *B. subtilis*, is the absence of *sdpABC* genes, which are involved in sporulation-delay followed by cannibalism [45]. However, three genes, *BASU_2886*-*BASU_2888* similar to the *sdp* operon were identified which may be a substitution for cannibalism in UCMB5113. Internalization of exogenous DNA (competence development) depends on several genes, some being expressed early during growth while others are activated later. The *B. amyloliquefaciens* subsp. *plantarum* UCMB5113 genome encodes all such genes as *comC, comK*, and *comEFG* operons, except the *comS* gene that is involved in the establishment of genetic competence [46], [47]. However, we uncovered three genes in the GI-06 region with substantially lower GC content (37%) that could putatively encode HsdS, HsdM, and HsdR subunits sharing significant identity to type-I restriction-modification systems.

### Sugar Transporters

*B. amyloliquefaciens* subsp. *plantarum* UCMB5113 encodes a plethora of distinct carrier proteins to transport the organic and inorganic nutrients available in the rhizosphere. Similarity searches and conserved domain analysis of protein sequences found categorizes of 430 putative carrier proteins including efflux and permeases (Table S4 in File S1). Out of this collection, 136 were ABC-type transporters (including the phosphate and zinc-specific operons *pst* and *znu*, respectively), 68 classified as MFS family transporters, 24 belonged to the phosphotransferase system (PTS) family of transporters, one putative membrane fusion protein (MFP), *BASU_1625*, belonging to the RND family of transporters. A member of the MMPL family with unknown function was also identified. It has been suggested that members of the MMPL family may be involved in lipid transport [48]. Presence of PTS transporters and other hydrolases/isomerases enable UCMB5113 to break down and utilize several plant-derived compounds as carbon sources. To investigate this, we tested the fermentation ability of UCMB5113 with several sugars. The sugars fermented by UCMB5113 are summarized in Table 3. In addition to common sugars this strain has the ability to metabolize acyclic polyols (sugar alcohols) such as sorbitol and D-mannitol by converting them to fructose via sorbitol dehydrogenase and mannitol-1-phosphate dehydrogenase. The UCMB5113 strain thus has a dynamic usage of different sugars although the differences in inducibility of sugar degradation capacity observed between 24 and 48 h, suggest preferences for certain types of sugars.

**Table 3:** Fermentation of sugars by UCMB5113 analyzed using API strips.

Substrate	Utilization	
	after 24 hrs	after 48 hrs
Glycerol	++++	++++
Erythritol	+	+
D-Arbinose	+	+
L-Arbinose	++++	++++
D-Ribose	++++	++++
D-Xylose	−	++
L-Xylose	−	+
D-Adonitol	−	+
Methyl-βD-Xylopyranoside	−	+
D-Galactose	+	+
D-Glucose	++++	++++
D-Fructose	++++	++++
D-Mannose	++++	++++
L-Sorbose	−	+
L-Rhamnose	−	+
Dulcitol	−	+
Inositol	++	++
D-Mannitol	++++	++++
D-Sorbitol	++++	++++
Methyl-αD-Mannopyranoside	−	+
Methyl-αD-Glucopyranoside	++	+++
N-Acetylglucosamine	−	−
Amygdalin	+++	++++
Arbutin	+++	v
Esculin, ferric citrate	++++	++++
Salicin	+++	+++
D-Cellobiose	++++	++++
D-Maltose	++++	++++
D-Lactose	++	+++
D-Melibiose	−	++
D-Saccharose	++++	+
D-Trehalose	++++	+
Inulin	−	−
D-Melizitose	−	−
D-Raffinose	−	+++
Amidon (starch)	−	+++
Glycogen	−	+++
Xylitol	−	+
Gentiobiose	+	+
D-Turanose	−	−
D-Lyxose	−	−
D-Tagatose	−	−
D-Fucose	−	−
L-Fucose	−	−
D-Arabitol	−	−
L-Arabitol	−	−
Potassium gluconate	−	−
Potassium 2-Ketogluconate	−	−
Potassium 5-Ketogluconate	−	−

++++: Highly positive reaction (Bright Yellow).

+++: Positive reaction (Dark Yellow).

++: Medium reaction (Bright Orange).

+: Weak reaction (Dark orange).

−: Negative (Red).

v: Variable reaction (positive to undetermined).

### Resistance to drugs and heavy metals

*B. amyloliquefaciens* subsp. *plantarum* UCMB5113 seems resistant to several antimicrobial compounds in the rhizosphere improving competitiveness in microbial antagonism and may also support establishment of a symbiotic relationship with the host plants. The genome encodes a putative tetB protein that contributes to tetracycline resistance by decreasing its accumulation in *B. subtilis* [49]. UCMB5113 growth on tetracycline containing LB plates confirmed this property with good growth recorded at 5 µg tetracycline ml^−1^. Another putative resistance gene, *yyaR*, was found displaying similarity to the *sat-4* gene that encodes a streptothricin acetyltransferase in *Campylobacter coli* BE/G4 [50]. Additionally, two novel genes (*BASU_2335* and *BASU_3689*) putatively encoding multidrug exporters were located that may also support resistance to several antibiotics.

The *B. amyloliquefaciens* subsp. *plantarum* UCMB5113 chromosome carries genes that also confer resistance to heavy metals such as zinc, copper, cobalt, and cadmium. UCMB5113 grown on LB plates containing up to 10 mM of cadmium, cobalt, copper or zinc exhibited good metal tolerance at all concentrations tested except for zinc (at 10 mM), and cadmium where some inhibition was noted at 1 and more at 10 mM concentration. The strain does not encode the genes *arsABC* involved in arsenic/arsenate resistance. However, a putative arsenic pump membrane protein YdfA share significant similarity to ArsB and may contribute to arsenic resistance [51], [52]. Altogether this property makes UCMB5113 a promising biocontrol agent candidate to serve also in metal contaminated soils or soils that by agricultural practices get higher metal content e.g, through addition of mud from sewage plants.

### Oxidative stress

*B. amyloliquefaciens* subsp. *plantarum* UCMB5113 seems capable to produce enzymes with proteolytic/hydrolytic like activities in response to oxidative stress in the rhizoplane, prior to colonization. Primarily, these enzymes include superoxide dismutases (SodA, SodC, SodF), three hydrogen peroxide decomposing catalases (KatA, KatE, KatX), manganese catalase (YdbD), three alkyl hydroperoxide reductases (AhpC and AhpF, BASU_0830), thiol peroxidase (tpx), glutathione peroxidase (gpo), bacillopeptidase F (bpr), gamma-glutamyl transpeptidase (ggt), and an operon (*ohrARB*) for resistance to organic peroxides [53]. Furthermore, the flavohemoprotein nitric oxide dioxygenase encoded by the genes *hmp* and *BASU_2738*, seem to protect the bacterium from nitrosative stress [54]. Accordingly, UCMB5113 has a battery of enzymes to prevent damage from reactive oxygen species that could be formed by non-enzymatic reactions in soil or the result of various biological processes including plant defense reactions at the colonization stage. The UCMB5113 battery of antioxidant genes could be of benefit also for the plant if such components are produced in the rhizosphere, e.g. as a result of abiotic stress, but quenched by the root colonizing bacteria.

### Swarming motility and chemotaxis

*B. amyloliquefaciens* subsp. *plantarum* UCMB5113 exhibits swarming motility (Figure 5). The presence of gene clusters (*flg, flh, fli*) for the production and assembly of functional flagellar components together with the *swrA* gene that up-regulates the expression of flagellar genes and increases swarming motility [55], [56] supports the high rhizosphere competence observed for UCMB5113. Production of lipopeptide biosurfactants is also an essential feature that facilitates motility by lowering the surface tension of solid surfaces changing interfacial interaction. In addition, genetic determinants of chemotaxis (*che*) were also identified in the genome. All these genes will greatly favour host plant sensing and identification to assure proper root colonization and formation of a suitable environment that enables proliferation of UCMB5113. A drop collapse test to evaluate the biosurfactant activity of UCMB5113 indeed showed that bacterial exudates caused a rapid loss of surface tension of aqueous drops indicating the presence of bioactive compounds (Figure 5).

### Biofilm formation and root colonization

Biofilm formation is essential for efficient surface colonization by bacteria, and this matrix is comprised of a variety of extracellular polymeric products. Exopolysaccharides are important components of the extracellular matrix making up the biofilm. Two operons, *epsA-O* and *yqxM-sipW-tasA*, that are required for the formation of robust biofilms in *B. subtilis* [57], [58], [59] were located in the UCMB5113 genome. Besides this, the protein YuaB is also probably encoded, which not only facilitates the assembly of a biofilm matrix [60], but also is responsible for biofilm surface repellency by forming a hydrophobic layer on the surface of the biofilm [61], which probably provides resistance to a broad spectrum of antimicrobial agents. In addition, *ypqP* probably encodes a capsular polysaccharide biosynthesis protein, with homology to CapD that has been implicated to epimerase UDP-galactose to UDP-glucose. This reaction contribute to the production of exopolysaccharide or lipopolysaccharide slime layers that surrounds the bacterium [62]. By contrast, an insertion of phage-related genes caused fragmentation of *ypqP* in the genome of *B. subtilis* 168 decreasing the biofilm production ability.

Root colonization by *B. amyloliquefacines* subsp. *plantarum* strains mainly determined by the chemotaxis and biofilm formation ability, is important for establishing associations with host plants, initiated after extensive and intricate cross talk. However, many steps in this process are not well understood thus far. Most probably factors like externalized polysaccharides, adhesins, and motility functions play significant roles in root colonization. Two of the *B. amyloliquefaciens* subsp. *plantarum* UCMB5113 genes, *BASU_0726* and *BASU_0727*, encode proteins with a collagen-like GXT structural motif expected to form extended fiber-like protein structures that may be involved in surface adhesion observed to occur for UCMB5113 on plant seeds and seedlings [10]. In addition, inhibition of the host innate immunity system or avoidance of recognition by plant's pattern recognition receptors is needed in order for UCMB5113 to avoid rejection from the plant.

### Plant growth promotion hormones and volatile compounds

**Figure 7:**
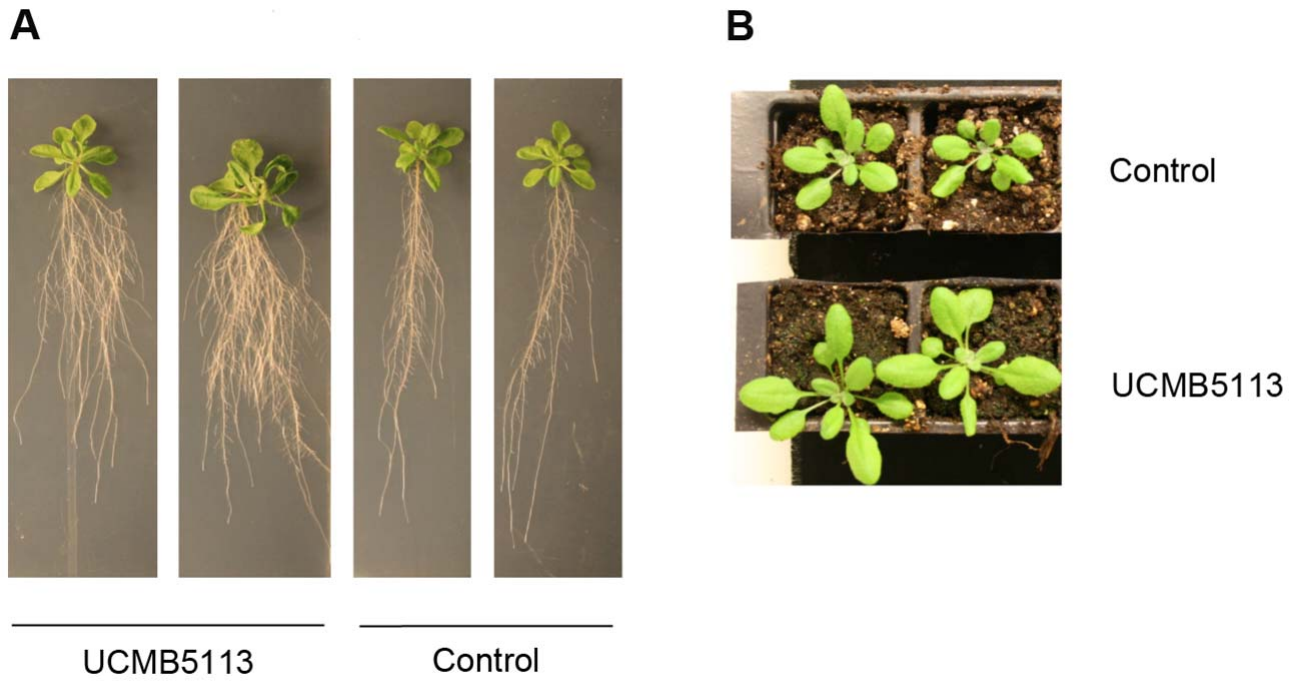
Plant growth promotion by UCMB5113 on *Arabidopsis thaliana* Col-0. (A). Plants grown on MS agar and treated with UCMB5113 display bigger leaves and increased root branching. (B) Plants grown on soil and treated with UCMB5113 have bigger leaves compared to control plants. The experiment was performed at least three times, and similar results were obtained in each case.

The production or stimulation of plant formation of phytohormones such as auxin, gibberillin, ethylene, abscisic acid, and cytokinin, is a characteristic feature of many PGPR. Plant-associated bacteria having the capacity to contribute to the host plants hormone pool can manipulate plant physiology and bring outcomes that favor their own survival. Auxin or indole-3-acetic acid (IAA) that serves as a master regulator of root growth and development is synthesized by several *Bacillus* species. Tryptophan dependent IAA synthesis involving putative IAA acetyltransferase (YsnE) and putative nitrilase (YhcX) and their effect on plant growth promotion has been demonstrated in *B. amyloliquefaciens* [63]. The presence of these two genes in the UMCB5113 genome tempts us to hypothesize that they play a prominent role in the enhanced plant growth observed when UCMB5113 is added to *Brassica napus* and *Arabidopsis thaliana* (Figure 7). Additionally, the gene *ywkB* putatively encoding an auxin efflux carrier protein that may be involved in the transport and redistribution of auxin to the roots was found in the UCMB5113 genome. However, the existance of intermediate compounds involved in IAA biosynthesis is not well known and has not been demonstrated for any *B. amyloliquefaciens* subsp. *plantarum* strain.

Volatile organic compounds (VOCs) like 3-hydroxy-2-butanone (acetoin) and 2,3-butanediol emitted by the rhizobacteria *B. subtilis* GB03 and *B. amyloliquefaciens* IN937a may not only trigger plant growth but a role in igniting ISR has also been implicated in plant-bacteria systems [64], [65]. Acetoin production in the two *Bacillus* strains involves two enzyme encoding genes, *alsS* and *alsD*, which encode acetolactate synthase and acetolactate decarboxylase, respectively. The biosynthesis of 2,3-butanediol is catalyzed by the enzyme (*R*,*R*)-butanediol dehydrogenase, which is encoded by *bdhA*. The UCMB5113 chromosome possesses genes that ferment pyruvate to acetoin and 2,3-butanediol. The growth promotion effect observed on plants by UCMB5113 can most likely be attributed to the action of these genes.

### Conserved ‘*plantarum*’ species coding genes

**Figure 8:**
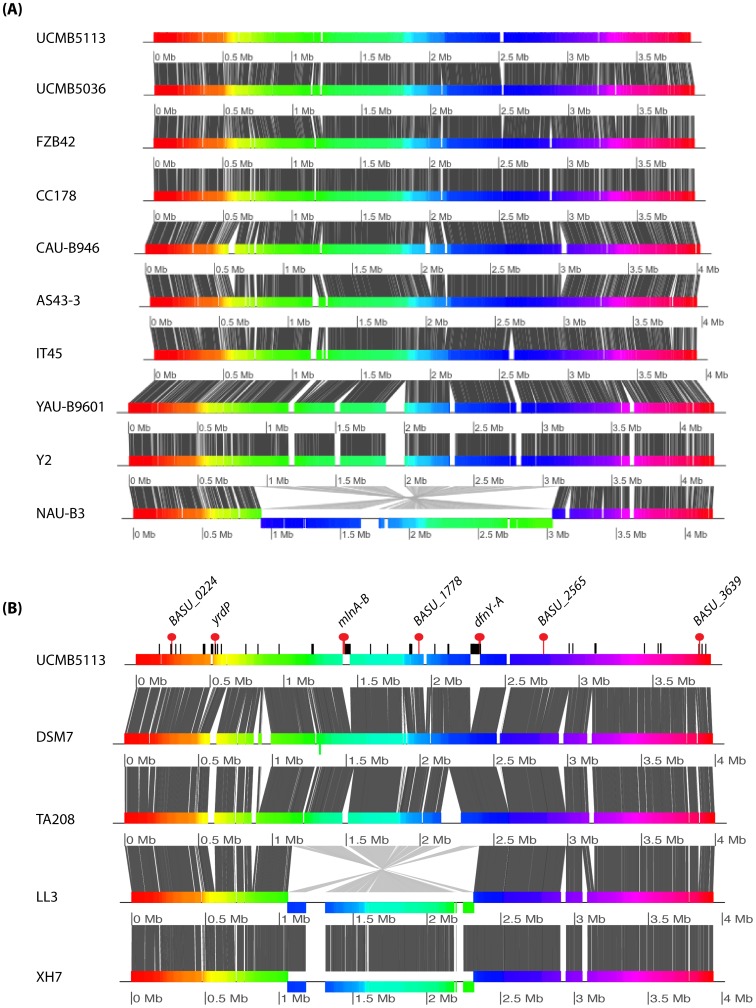
Genomic overview of the similarity between completely sequenced *B. amyloliquefaciens* strains. (A) Genomic comparison map of plant-associated *B. amyloliquefaciens* subsp. *plantarum* strains. The grey blocks indicate similarity and sequence conservation whereas gaps between the blocks show differences in genomic content between genomes. The rainbow color bar shows synteny between genomes. (B) Comparison of the plant growth promoting strain UCMB5113 (as representative of *plantarum* species) with non plant-associated subsp. *amyloliquefaciens* strains. Vertical bars (black and red) on the top show the location of *plantarum* group specific genes in UCMB5113, whereas red dots indicate nine of the selected *plantarum*-specific genes that were shown to be expressed (Figure S2).

Gene content comparison of the two groups of *B. amyloliquefaciens* species performed using the proteome of *B. amyloliquefaciens* subsp. *plantarum* UCMB5113 as reference, identified characteristic features of ‘*plantarum*’ species (Figure 8). Interestingly, two large genomic regions of size 70 kb and 53 kb (occupied by the *dfn* and *mln* gene clusters, respectively) were found to be conserved only in the genomes of plant-associated species. Conservation of *dfn* and *mln* gene clusters strongly suggests their role is not confined only to antimicrobial activities but may be linked with plant-associated activities as to become accepted as a beneficial partner since certain polyketides have been indicated to serve both as virulence and avirulence factors [66]. In total, 80 protein-coding genes were identified with no counterparts in the genomes of the strains belonging to the *B*. *amyloliquefaciens* subsp. *amyloliquefaciens* group (Table S5 in File S1). The identified genetic elements may represent characteristics of the plantarum species i.e. host plant association and rhizosphere competence. Results from expression analysis of a selection of nine of these genes confirmed that the predicted genes were indeed expressed (Figure S2), and thus may have role for plant interaction.

## Conclusions

The genome assembly of *B. amyloliquefaciens* subsp. *plantarum* UCMB5113 and its comparison with the genomes of model species of *B. subtilis* group gave solid basis for gene annotation. The UCMB5113 strain seems to have high capacity to produce different kinds of antibiotics and also secrete a large number of enzymes to improve nutrient acquisition in the rhizosphere. This strain also seems to have potential for production of hormones and volatile compounds that support plant growth and thereby improve plant roots and biomass improving plant quality as a colonization partner and at the same time also increase surface area for colonization and the nutrient resource for the bacteria. UCMB5113 seems able to use a wide range of sugars and other organic compounds that could be present in root exudates. In return the bacteria antagonize detrimental soil microorganisms and strengthen plants improving their nutrient and stress handling capabilities. The ability of UCMB5113 to quench reactive oxygen species should be beneficial for the plant to decrease stress damage. The annotation of UCMB5113 from this study will pave way in elucidating the mechanism involved in plant-bacterial interation. Comparison with other related *B. amyloliquefaciens* genomes that differ in their capability of plant colonization, growth promotion and stress tolerance provides an excellent basis for *in silico* predictions of gene candidates and regulatory factors that are involved in these processes. We are currently searching for plant genotypes that vary in the interaction with *Bacillus* strains as a basis to pinpoint plant genes important for the interactions. Deciphering of the molecular determinants for these processes open up possibilities to identify even more efficient *Bacillus* strains, engineer improved strains, and optimize conditions that favour interaction and colonization to support durable crop production.

## Supporting Information

Figure S1 **Global alignment of bacterial chromosomes.** Shows highly conserved regions between the genomes of *B. amyloliquefaciens* subsp. *plantarum* UCMB5113, *B. amyloliquefaciens* DSM7, *B. subtilis* 168 and *B. pumilus* SARF-032
(TIF)

Figure S2 **Expression analysis of UCMB5113 genes specific to** ***plantarum*** **species.** The genes expressed during the exponential growth phase of UCMB5113. Each lane was loaded with 5ul of RT-PCR amplified product. The *tetB* gene was used as an expression control.
(TIF)

File S1 **Contains the files**: **Table S1**: Regions of genomic plasticity (RGP) in *Bacillus amyloliquefaciens* UCMB5113 genome. **Table S2.** Deletions occurring in the UCMB5113 genome in comparison to strain FZB42. In some cases deletions were partially substituted by RGPs or smaller insertions. **Table S3**: Putative CDS with secretory signal peptides in *B. amyloliquefaciens* UCMB5113 genome. **Table S4**: List of Transporter proteins encoded by *B. amyloliquefaciens* UCMB5113. **Table S5**. List of *plantarum* species-specific genes.
(DOCX)
