# Supplementary figures and images for: Genome Analysis of Bacillus amyloliquefaciens Subsp. plantarum UCMB5113: A Rhizobacterium That Improves Plant Growth and Stress Management
Source: PLoS One. 2014 Aug 13;9(8):e104651. doi: 10.1371/journal.pone.0104651 (PMC4138018; doi:10.1371/journal.pone.0104651)

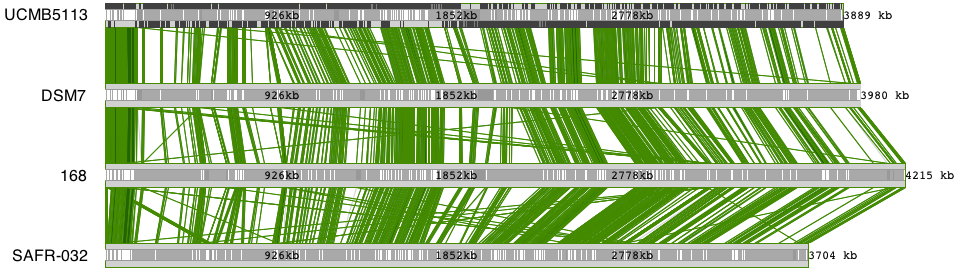

Supplement: Figure S1 — Global alignment of bacterial chromosomes. Shows highly conserved regions between the genomes of B. amyloliquefaciens subsp. plantarum UCMB5113, B. amyloliquefaciens DSM7, B. subtilis 168 and B. pumilus SARF-032 (TIF) [file pone.0104651.s001.tif]

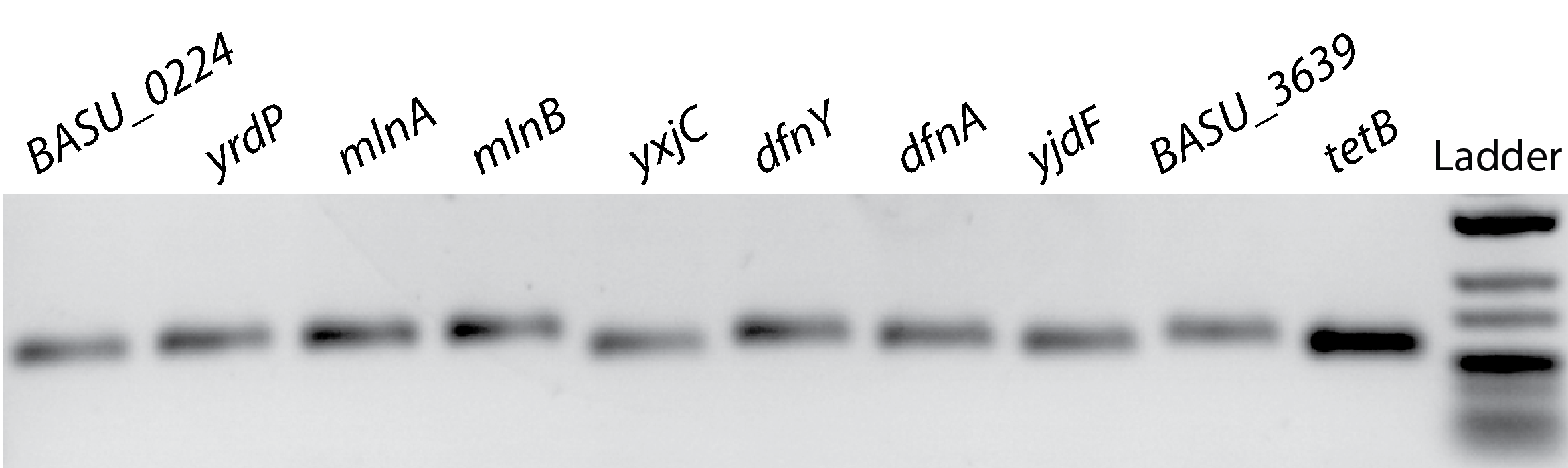

Supplement: Figure S2 — Expression analysis of UCMB5113 genes specific to plantarum species. The genes expressed during the exponential growth phase of UCMB5113. Each lane was loaded with 5ul of RT-PCR amplified product. The tetB gene was used as an expression control. (TIF) [file pone.0104651.s002.tif]
