# Supplementary material for: Genome Analysis of Bacillus amyloliquefaciens Subsp. plantarum UCMB5113: A Rhizobacterium That Improves Plant Growth and Stress Management
Source: PLoS One. 2014 Aug 13;9(8):e104651. doi: 10.1371/journal.pone.0104651 (PMC4138018; doi:10.1371/journal.pone.0104651)
Supplement: File S1 — Contains the files: Table S1: Regions of genomic plasticity (RGP) in Bacillus amyloliquefaciens UCMB5113 genome. Table S2. Deletions occurring in the UCMB5113 genome in comparison to strain FZB42. In some cases deletions were partially substituted by RGPs or smaller insertions. Table S3: Putative CDS with secretory signal peptides in B. amyloliquefaciens UCMB5113 genome. Table S4: List of Transporter proteins encoded by B. amyloliquefaciens UCMB5113. Table S5. List of plantarum species-specific genes. (DOCX) [file pone.0104651.s003.docx]

**Figure S1.** **Global alignment of bacterial chromosomes.** Shows highly conserved regions between the genomes of *B. amyloliquefaciens* subsp. *plantarum* UCMB5113, *B. amyloliquefaciens* DSM7, *B. subtilis* 168 and *B. pumilus* SARF-032

**Figure S2.** **Expression analysis of UCMB5113** **genes specific to *plantarum* species.** The genes expressed during the exponential growth phase of UCMB5113. Each lane was loaded with 5ul of RT-PCR amplified product. The *tetB* gene was used as an expression control.

**Table S1:** Regions of genomic plasticity (RGP) in *Bacillus* *amyloliquefaciens* UCMB5113 genome.

|  |  | |  | |  |  | | | |  |  |  |  |  |  |  | **Specificity score %** | |
| --- | --- | --- | --- | --- | --- | --- | --- | --- | --- | --- | --- | --- | --- | --- | --- | --- | --- | --- |
| **Label** | **Start** | | **End** | | **Length** | **Key features** | | | | **ORFs** | **Unique** | **Phage**  **genes** | **GC** | **IS/int/**  **resolvase** | **tRNA/**  **misc_RNA** | **Prediction Program** | **FZB42** | ***B. subtilis 168*** |
| RGP1a | 206594 | | 213120 | | 6527 | ABC transporters, ribosomal proteins, methyltransferases | | | | 6 | 3 | **-** | **+** | **-** | **-** | SIGI, IVOM,  RGP Finder | 50 | 100 |
| RGP1b | 215350 | | 223515 | | 8166 | permeases, kinases, hydrolases, phage proteins, ABC transporters | | | | 9 | 0 | **+** | **-** | **-** | **-** | RGP Finder | 0 | 56 |
| RGP2 | 457031 | | 462338 | | 5308 | acetyltransferases, racemases, phage rerlated proteins | | | | 11 | 3 | **+** | **+** | **-** | **-** | IVOM, RGP Finder | 73 | 64 |
| RGP3a | 496632 | | 555846 | | 59215 | tRNAs, peptidases, doxX, dehyderatases, racemases, NADPH nitroreductase, NADP oxidoreductase | | | | 75 | 7 | **-** | **+** | **-** | **+** | SIGI, IVOM,  RGP Finder | 31 | 71 |
| RGP3b | 558616 | | 579789 | | 21174 | acetyltransferases, dehydrogenases, methyltransferase, ABC transporters | | | | 25 | 2 | **-** | **-** | **-** | **-** | RGP Finder | 8 | 60 |
| RGP4 | 609680 | | 618907 | | 9228 | endonuclease, type-1 restriction-modification system | | | | 6 | 4 | **-** | **+** | **-** | **-** | SIGI, IVOM,  RGP Finder | 50 | 100 |
| RGP5 | 694433 | | 701695 | | 7263 | keto-acyl carrier proteins, oxoacyl carrier proteins, redecutases, ATPase | | | | 9 | 7 | **-** | + | **-** | **-** | SIGI, IVOM,  RGP Finder | 89 | 100 |
| RGP6 | 748838 | | 754007 | | 5170 | Collagen-like triple helix with GXT repeats | | | | 5 | 1 | **-** | **-** | **-** | **-** | IVOM, RGP Finder | 40 | 100 |
| RGP7 | 870282 | | 875887 | | 5606 | Oligopeptide permease system | | | | 5 | 5 | **-** | **-** | **-** | **-** | RGP Finder | 20 | 20 |
| RGP8 | 1153248 | | 1175838 | | 22591 | tRNA, secA, Arabinogalactan endo-1,4 beta galactosidase, Galactose-1-phosphate uridylyltransferase, Lactose PTS system | | | | 24 | 6 | **-** | **+** | **-** | **+** | IVOM, RGP Finder | 29 | 92 |
| RGP9 | 1207715 | | 1225874 | | 18159 | Prophage | | | | 21 | 0 | **+** | **-** | **-** | **-** | RGP Finder | 100 | 0 |
| RGP10 | 1262173 | | 1267690 | | 5518 | glycosyl transferase, phenylpropionic acid transporter | | | | 6 | 0 | **-** | **-** | **-** | **-** | RGP Finder | 0 | 100 |
| RGP11 | 1317725 | | 1323681 | | 5956 | oxidoreductase, hydrolase | | | | 10 | 0 | **-** | **+** | **-** | **-** | SIGI, IVOM | 30 | 10 |
| RGP12 | 1395814 | | 1449067 | | 53254 | macrolactin biosynthesis | | | | 9 | 0 | **-** | **-** | **-** | **-** | RGP Finder | 0 | 89 |
| RGP13 | 1806105 | | 1827522 | | 21418 | alcohol dehydrogenase, acetyltransferase, chitin binding | | | | 19 | 4 | **+** | **+** | **-** | **-** | SIGI, IVOM,  RGP Finder | 26 | 100 |
| RGP14 | 1874082 | | 1911362 | | 37281 | bacillomycin bmy biosynthesis | | | | 5 | 1 | **-** | **-** | **-** | **-** | IVOM, RGP Finder | 20 | 60 |
| RGP15 | 2260807 | | 2333264 | | 72458 | dfn biosynthesis | | | | 18 | 0 | - | - | - | - | RGP Finder | 0 | 72 |
| RGP16 | 2502670 | | 2545483 | | 42814 | tetB, old, Putative nrps/pks biosynthesis, t-box | | | | 34 | 17 | **+** | **+** | **+** | **+** | SIGI, IVOM,  RGP Finder | 68 | 94 |
| RGP17 | 2801930 | | 2814498 | | 12569 | aldehyde dehydrogenase, nitric oxide dioxygenase | | | | 19 | 5 | **+** | **+** | **-** | **-** | SIGI, IVOM,  RGP Finder | 29 | 100 |
| RGP18 | 3037652 | | 3043759 | | 6108 | ABC transporter, N-acetyltransferase, Putative bacteriocin uberolysin | | | | 8 | 0 | **-** | **+** | **-** | **-** | IVOM, RGP Finder | 0 | 100 |
| RGP19 | 3218087 | | 3228696 | | 10610 | xylose isomerase, N-acetylglucosaminyl phosphatidylinositol deacetylase | | | | 10 | 0 | **-** | **-** | **-** | **-** | RGP Finder | 0 | 100 |
| RGP20 | 3278782 | | 3291076 | | 12295 | non-PTS sugar transport system | | | | 16 | 1 | **-** | **+** | **-** | **+** | RGP Finder | 14 | 86 |
| RGP21 | 3567816 | | 3573387 | | 5572 | ABC transporters | | | | 8 | 0 | **-** | **+** | **-** | **-** | RGP Finder | 0 | 75 |
| RGP22a | 3780938 | | 3789243 | | 8306 | glycine, transporters, reductases and dehydrogenases | | | | 7 | 0 | **-** | **-** | **-** | **+** | RGP Finder | 14 | 71 |
| RGP22b | 3794631 | | 3801358 | | 6728 | Unknown proteins | | | | 7 | 6 | **-** | **+** | **-** | **-** | IVOM, RGP Finder | 86 | 100 |
| RGP23 | 3814429 | | 3820090 | | 5662 | ABC transporter, Putative quinolone resistance protein, kinase, signal transduction | | | | 6 | 0 | **-** | **-** | **-** | **-** | RGP Finder | 0 | 67 |
| RGP24a | 3833076 | | 3839356 | | 6281 | glycosyl transferase, starch/glycogen synthase, N-acetylglucosaminyl phosphatidylinositol deacetylase | | | | 6 | 0 | **-** | **-** | **-** | **-** | RGP Finder | 0 | 100 |
| RGP24b | 3845532 | | 3850578 | | 5047 | permease, levansucrase, endolevanase | | | | 4 | 0 | **-** | **-** | **-** | **-** | RGP Finder | 0 | 50 |
| RGP24c | 3855878 | | 3864477 | | 8600 | Putative 4-carboxymuconolactone decarboxylase, acetyltransferase, 3-hydroxybutyrate dehydrogenase | | | | 9 | 0 | **-** | **+** | **-** | **-** | SIGI, IVOM,  RGP Finder | 22 | 56 |
|  | |  | |  | | |  |  |  |  |  |  |  |  |  |  |  |  |

**Abbreviations:** IS: insertion sequence; int: integrase gene; GC: GC% deviation; RGP, Region of Genomic Plasticity "sensu stricto". The specificity score indicates percentage of genes missing in FZB42 and *B. subtils* 168 from ORFs found in UCMB5113.

**Table S2.** Deletions occurring in the UCMB5113 genome in comparison to strain FZB42. In some cases deletions were partially substituted by RGPs or smaller insertions.

| **Deletions** | **FZB42** | | |  | **UCMB5113** | | |
| --- | --- | --- | --- | --- | --- | --- | --- |
|  | **From** | **To** | **Size (bp)** |  | **From** | **To** | **Size (bp)** |
| D1 | 311266 | 312131 | 864 |  | 308872 | 309041 | 168 |
| D2 | 503645 | 507057 | 3411 |  | 509888 | 509889 | 0 |
| D3 | 517204 | 519492 | 2287 |  | 520064 | 520065 | 0 |
| D4 | 523023 | 528084 | 5060 |  | 523579 | 523746 | 166 |
| D5 | 540794 | 542344 | 1549 |  | 536584 | 536641 | 56 |
| D6 | 556887 | 560115 | 3227 |  | 551189 | 551251 | 61 |
| D7 | 567236 | 571629 | 4392 |  | 558361 | 558525 | 163 |
| D8 | 621439 | 626029 | 4589 |  | 609448 | 619048 | 9599* |
| D9 | 698917 | 708564 | 9646 |  | 694310 | 703696 | 9385* |
| D10 | 709028 | 710236 | 1207 |  | 704160 | 704339 | 178 |
| D11 | 726188 | 736402 | 10213 |  | 721329 | 721330 | 0 |
| D12 | 798125 | 798942 | 816 |  | 782744 | 782745 | 0 |
| D13 | 830714 | 833520 | 2805 |  | 814534 | 814666 | 131 |
| D14 | 1042839 | 1043565 | 725 |  | 1029308 | 1029309 | 0 |
| D15 | 1164207 | 1169835 | 5627 |  | 1153322 | 1153323 | 0 |
| D16 | 1172673 | 1175283 | 2609 |  | 1156159 | 1156251 | 91 |
| D17 | 1190154 | 1192280 | 2125 |  | 1173903 | 1175167 | 1263 |
| D18 | 1335799 | 1337095 | 1295 |  | 1341014 | 1341015 | 0 |
| D19 | 1458644 | 1461184 | 2539 |  | 1462608 | 1462609 | 0 |
| D20 | 1792850 | 1795129 | 2278 |  | 1796538 | 1796649 | 110 |
| D21 | 1808652 | 1809249 | 596 |  | 1810233 | 1810234 | 0 |
| D22 | 1812042 | 1812215 | 172 |  | 1814488 | 1815816 | 1327 |
| D23 | 1812755 | 1813375 | 619 |  | 1816351 | 1816352 | 0 |
| D24 | 1860767 | 1861837 | 1069 |  | 1864374 | 1864918 | 543 |
| D25 | 1870766 | 1871200 | 433 |  | 1873831 | 1874111 | 279 |
| D26 | 1991754 | 1995772 | 4017 |  | 1994677 | 1994761 | 83 |
| D27 | 2078470 | 2094520 | 16049 |  | 2079169 | 2079170 | 0 |
| D28 | 2134479 | 2135346 | 866 |  | 2119361 | 2119423 | 61 |
| D29 | 2522466 | 2529986 | 7519 |  | 2505335 | 2538221 | 32885* |
| D30 | 2791430 | 2796698 | 5267 |  | 2804065 | 2807322 | 3256 |
| D31 | 2868278 | 2887890 | 19611 |  | 2880837 | 2881019 | 181 |
| D32 | 3115335 | 3117894 | 2558 |  | 3110309 | 3110318 | 8 |
| D33 | 3190774 | 3194514 | 3739 |  | 3183190 | 3183191 | 0 |
| D34 | 3254063 | 3259036 | 4972 |  | 3244506 | 3244765 | 258 |
| D35 | 3453335 | 3458875 | 5539 |  | 3443391 | 3444377 | 985 |
| D36 | 3609045 | 3613820 | 4774 |  | 3594566 | 3595214 | 647 |
| D37 | 3639935 | 3641224 | 1288 |  | 3621327 | 3621328 | 0 |
| D38 | 3763484 | 3764775 | 1290 |  | 3747827 | 3747828 | 0 |
| D39 | 3769583 | 3774824 | 5240 |  | 3752636 | 3752739 | 102 |
| D40 | 3840959 | 3843086 | 2126 |  | 3818376 | 3818466 | 89 |
| D41 | 3878853 | 3881045 | 2191 |  | 3854182 | 3854190 | 7 |
| D42 | 3888526 | 3893672 | 5145 |  | 3861690 | 3864648 | 2957 |

*Region of genome plasticity (RGP) substitutes the deletion.

**Table S3:** Putative CDS with secretory signal peptides in *B. amyloliquefaciens* UCMB5113 genome

**Genes predicted to contain Signal Peptidase I (SPI) cleavage sites**

| **Label** | **Unique/Exp** | **THMM2** | **Product** |
| --- | --- | --- | --- |
| BASU_0012_dacA |  | 0 | D-alanyl-D-alanine carboxypeptidase (penicillin-binding protein 5) |
| BASU_0076_yacD |  | 1 | Putative protein secretion prsa homolog |
| BASU_0179_ybbC |  | 1 | Uncharacterized protein ybbc |
| BASU_0180_nagZ |  | 0 | N-acetylglucosaminidase lipoprotein |
| BASU_0181_amiE |  | 0 | Amidase hydrolyzing N-acetylmuramyl-L-Ala bond of murnac peptides |
| BASU_0189_ybbR |  | 1 | Ybbr-like domain-containing protein ybbr |
| BASU_0209_yolA1 | exp. verified | 1 | Spbc2 prophage-derived uncharacterized protein yola |
| BASU_0217_glpQ | exp. verified | 1 | Gplq (glycerophosphoryl diester phosphodiesterase) |
| BASU_0224 |  | 1 | Conserved exported protein of unknown function |
| BASU_0258_estA | exp. verified | 2 | Secreted alkaliphilic lipase |
| BASU_0285_amyE | exp. verified | 1 | Amye alpha-amylase |
| BASU_0289_ycgF |  | 6 | Putative aminoacid export permease |
| BASU_0294_lci |  | 0 | Antimicrobial peptide, Lci |
| BASU_0316_yckD |  | 1 | Conserved hypothetical exported protein of unknown function |
| BASU_0319_nucA |  | 1 | Endonuclease |
| BASU_0348_yxeK |  | 0 | Putative monooxygenase |
| BASU_0356_yclI |  | 4 | Putative transporter |
| BASU_0360_phrC |  | 1 | Secreted regulator of the activity of phosphatase rapc and competence and sporulation stimulating factor (CSF) |
| BASU_0377_ycnI |  | 2 | Uncharacterized protein ycni |
| BASU_0378_ycnJ |  | 9 | Putative copper import protein |
| BASU_0394_yczI |  | 3 | Uncharacterized protein yczi |
| BASU_0410_ydaL |  | 2 | Uncharacterized protein ydal |
| BASU_0412_ydaN |  | 2 | Uncharacterized protein ydan |
| BASU_0429_A2 |  | 2 | Putative antifungal polypeptide |
| BASU_0438_dctB |  | 1 | C4-dicarboxylate binding protein |
| BASU_0558 | unique | 1 | Exported protein of unknown function |
| BASU_0566 | unique | 2 | Conserved exported protein of unknown function |
| BASU_0605_yjdJ |  | 2 | Uncharacterized protein yjdj |
| BASU_0612_ydjH |  | 2 | UPF0603 protein ydjh |
| BASU_0614_ydjM |  | 0 | Putative prc mutant suppressor rlpa |
| BASU_0615_ydjN |  | 1 | Putative membrane protein |
| BASU_0646_yezF |  | 2 | Hypothetical protein yezf |
| BASU_0721_pel | exp. verified | 0 | Pectate lyase pel |
| BASU_0753_yfkN | exp. verified | 2 | Trifunctional nucleotide phosphoesterase protein [Includes: 2',3'-cyclic-nucleotide 2'-phosphodiesterase/3'-nucleotidase ; 5'-nucleotidase] |
| BASU_0761_yfkJ |  | 0 | Protein-tyrosine-phosphatase |
| BASU_0767_yfkD |  | 0 | Conserved hypothetical protein |
| BASU_0771_pdaA |  | 0 | Probable polysaccharide deacetylase pdaa |
| BASU_0812_mpr | exp. verified | 0 | Extracellular glutamyl endopeptidase |
| BASU_0814_yfhK |  | 1 | Uncharacterized protein with a bacterial SH3 domain homologue/General stress protein, similar to cell division inhibitor |
| BASU_0870_yhcC | exp. verified | 1 | Hypotheticalprotein yhcc |
| BASU_0884_yhcR | exp. verified | 1 | Extracellular 5'-nucleotidase |
| BASU_0901_yhdC |  | 0 | Putative exported protein |
| BASU_0902_lytF |  | 0 | Gamma-D-glutamate-meso-diaminopimelate muropeptidase (major autolysin) |
| BASU_0906_phoA | exp. verified | 0 | Alkaline phosphatase A |
| BASU_0907_lytE |  | 1 | Lyte |
| BASU_0928_yheN |  | 1 | Putative polysaccharide deacetylase |
| BASU_0948_khtS |  | 0 | Putative K+/H+ antiporter for K+ efflux |
| BASU_0962_yhaH |  | 1 | Putative membrane protein; acid tolerance protein |
| BASU_0989_aprE | exp. verified | 1 | Serine alkaline protease (subtilisin E) |
| BASU_1011_yhjG |  | 0 | Putative aromatic compound monooxygenase/hydroxylase |
| BASU_1056_yitY |  | 1 | Putative FMN/FAD-binding oxidoreductase |
| BASU_1073_fabF |  | 0 | Beta-ketoacyl-acyl-carrier-protein synthase II |
| BASU_1087 |  | 0 | Conserved exported protein of unknown function |
| BASU_1125_blm |  | 0 | Beta-lactamase 2 |
| BASU_1142_penP | exp. verified | 1 | Beta-lactamase precursor |
| BASU_1144_yfjF |  | 14 | Yfjf |
| BASU_1146_ganA | exp. verified | 1 | Gana |
| BASU_1180_phrA |  | 1 | Secreted inhibitor of the activity of phosphatase rapa |
| BASU_1232_yubD |  | 14 | Putative efflux transporter |
| BASU_1321_ykvT |  | 0 | Cell wall hydrolase related to spore cortex-lytic enzymes |
| BASU_1323_stoA |  | 0 | Thiol-disulfide isomerase |
| BASU_1335_ykwD |  | 0 | Conserved hypothetical protein |
| BASU_1369_moaD |  | 0 | Molybdopterin synthase (small subunit) |
| BASU_1416_nprE | exp. verified | 0 | Extracellular neutral metalloprotease |
| BASU_1417_ylaE |  | 0 | Conserved exported protein of unknown function |
| BASU_1446_ylbJ |  | 8 | Putative factor required for spore cortex formation |
| BASU_1469_bpr | exp. verified | 1 | Bacillopeptidase F |
| BASU_1497_cysP |  | 8 | Sulfate permease |
| BASU_1509_coaBC |  | 0 | Coenzyme A biosynthesis bifunctional protein coabc; phosphopantothenoylcysteine synthetase/decarboxylase |
| BASU_1523_yloV |  | 0 | Putative dihydroxyacetone/glyceraldehyde kinase |
| BASU_1570_fliL |  | 1 | Flagellar basal-body associated protein |
| BASU_1574_fliZ |  | 2 | Flagellar regulatory protein |
| BASU_1610_ylxY |  | 1 | Putative sugar deacetylase |
| BASU_1635_pbpX | exp. verified | 1 | Penicillin-binding endopeptidase X |
| BASU_1664_nucB |  | 1 | Nuclease |
| BASU_1709_yoaO | unique | 1 | Conserved exported protein hypothetical protein |
| BASU_1711_chbA | exp. verified | 0 | Putative chitin binding protein |
| BASU_1720 |  | 0 | Conserved hypothetical protein |
| BASU_1749_ynfC |  | 2 | Conserved hypothetical protein |
| BASU_1760_yndJ |  | 8 | Putative integral inner membrane protein |
| BASU_1764_eglS | exp. verified | 1 | Endo-1,4-beta-glucanase |
| BASU_1769_xynC | exp. verified | 1 | Endo-xylanase |
| BASU_1770_xynD | exp. verified | 0 | Arabinoxylan arabinofuranohydrolase |
| BASU_1786_yngB |  | 0 | Putative UTP-glucose-1-phosphate uridylyltransferase |
| BASU_1795_yngK |  | 1 | Putative exported protein |
| BASU_1802_dacC |  | 0 | D-alanyl-D-alanine carboxypeptidase |
| BASU_1805_iseA |  | 0 | Inhibitor of cell-separation enzymes |
| BASU_1808_ggt | exp. verified | 1 | Membrane bound gamma-glutamyltranspeptidase |
| BASU_1821 |  | 1 | Conserved exported protein of unknown function |
| BASU_1845_yocH | exp. verified | 0 | Putative exported cell wall-binding protein |
| BASU_1867_cwlS |  | 0 | Peptidoglycan hydrolase (cell wall-binding d,l-endopeptidase) |
| BASU_1886 |  | 1 | Glucan endo-1,6-Î²-glucosidase |
| BASU_1889_ctpA |  | 1 | Carboxy-terminal processing protease |
| BASU_1914_phy | exp. verified | 0 | Phytase |
| BASU_1919_ypmS |  | 1 | Conserved hypothetical protein |
| BASU_1929_ypjP |  | 1 | Uncharacterized protein ypjp |
| BASU_1955 | unique | 0 | Conserved exported protein of unknown function |
| BASU_1980_aspB |  | 0 | Putative aspartate aminotransferase |
| BASU_1981_ypmB |  | 1 | Conserved hypothetical protein |
| BASU_1996_ypjB |  | 1 | Spore formation membrane associated protein |
| BASU_2027_gpsA |  | 0 | NAD(P)H-dependent glycerol-3-phosphate dehydrogenase |
| BASU_2036_sleB |  | 0 | Spore cortex-lytic enzyme |
| BASU_2041_ypbG |  | 0 | Putative phosphoesterase |
| BASU_2048_fmnP |  | 5 | FMN permease |
| BASU_2056_resA |  | 1 | Extracytoplasmic thioredoxin involved in cytochrome c maturation (lipoprotein) |
| BASU_2060_dacB |  | 0 | D-alanyl-D-alanine carboxypeptidase (penicillin-binding protein 5*) |
| BASU_2071_ypuD |  | 1 | Conserved exported protein of unknown function |
| BASU_2075_ypuA |  | 0 | Putative exported protein |
| BASU_2087_dacF |  | 0 | D-alanyl-D-alanine carboxypeptidase (penicilin binding protein) |
| BASU_2098_ansA |  | 0 | Exported L-asparaginase |
| BASU_2104_yqkD |  | 1 | Putative hydrolase |
| BASU_2145_oxaAB |  | 5 | Sec-independent factor for membrane protein insertion (yidc/spoiiij family) |
| BASU_2149_yqjB |  | 0 | Putative LD-transpeptidase yqjb |
| BASU_2169_mmgB |  | 0 | 3-hydroxybutyryl-coa dehydrogenase |
| BASU_2172_yqiI |  | 0 | Putative N-acetylmuramoyl-L-alanine amidase |
| BASU_2197_spoIIIAE |  | 7 | Stage III sporulation protein |
| BASU_2219_tasA | exp. verified | 1 | Major biofilm matrix component |
| BASU_2221_yqxM |  | 1 | Protein for biofilm formation |
| BASU_2223_yqzG |  | 0 | Putative exported protein |
| BASU_2248_yqzC |  | 1 | Putative sporulation membrane protein |
| BASU_2259_yqfZ |  | 1 | Factor involved in motility |
| BASU_2293_yqeZ |  | 5 | Putative membrane bound hydrolase |
| BASU_2326 |  | 0 | Putative polysaccharide deacetylase |
| BASU_2353_yqaC |  | 0 | Putative kinase; skin element |
| BASU_2383_yrrS |  | 1 | Conserved hypothetical protein |
| BASU_2384_pbpI |  | 1 | Penicillin-binding protein PBP4B |
| BASU_2412_yrvJ |  | 1 | Putative N-acetylmuramoyl-L-alanine amidase family 3 |
| BASU_2430_bofC |  | 1 | Bypass of forespore C intercompartmental signaling factor |
| BASU_2436_nadB |  | 0 | L-aspartate oxidase |
| BASU_2468_ysxD |  | 6 | Putative integral inner membrane protein |
| BASU_2494_sdhA |  | 0 | Succinate dehydrogenase (flavoprotein subunit) |
| BASU_2530_abnA | exp. verified | 1 | Arabinan-endo 15-alpha-L-arabinase |
| BASU_2558_phoR |  | 2 | Two-component sensor histidine kinase |
| BASU_2560_mdh |  | 0 | Malate dehydrogenase |
| BASU_2568_ytvI |  | 8 | Putative permease |
| BASU_2610 | unique | 0 | Exported protein of unknown function |
| BASU_2735_yuaB | exp. verified | 1 | Biofilm assembly factor |
| BASU_2737_lytG |  | 1 | Exoglucosaminidase |
| BASU_2740_yjcN |  | 1 | Uncharacterized exported protein yjcn |
| BASU_2748_tlpA |  | 2 | Methyl-accepting chemotaxis protein |
| BASU_2761_yugM |  | 1 | Uncharacterized membrane protein yugm |
| BASU_2779_pbpD |  | 1 | Penicillin-binding protein 4 |
| BASU_2782_yufL |  | 2 | Two-component sensor histidine kinase [yufm] |
| BASU_2813_yueC |  | 2 | Uncharacterized exported protein yuec |
| BASU_2841 |  | 5 | Uncharacterized membrane protein |
| BASU_2844_ublA |  | 2 | Circular bacteriocin (circularin A/uberolysin) |
| BASU_2865_lytH |  | 0 | Sporulation-specific L-Ala-D-Glu endopeptidase |
| BASU_2877_bsn | exp. verified | 1 | Extracellular ribonuclease |
| BASU_2886 | unique | 2 | Putative killing factor like sdpc/Toxic peptide sdpc |
| BASU_2891_yurR |  | 0 | Putative oxidoreductase |
| BASU_2894_csn | exp. verified | 1 | Chitosanase |
| BASU_2913_fadN |  | 0 | Enoyl-coa hydratase / 3-hydroxyacyl-coa dehydrogenase |
| BASU_2927_yusZ |  | 0 | Putative short-chain acyl dehydrogenase |
| BASU_2942_liaG |  | 0 | Protein liag |
| BASU_2976_yvgO | exp. verified | 0 | Stress response protein yvgo |
| BASU_2982 |  | 0 | Metalloproteinase |
| BASU_2984_bdbD |  | 1 | Thiol-disulfide oxidoreductase |
| BASU_3003 | unique | 3 | Uncharacterized exported membrane protein |
| BASU_3041_ywaD | exp. verified | 0 | Aminopeptidase ywad |
| BASU_3051 | unique | 0 | Conserved exported protein of unknown function |
| BASU_3055 | unique | 1 | Conserved exported protein of unknown function |
| BASU_3070_epsL |  | 1 | Putative phosphotransferase involved in extracellular matrix synthesis |
| BASU_3108 |  | 1 | Ribonuclease |
| BASU_3117_cwlO | exp. verified | 0 | Secreted cell wall DL-endopeptidase |
| BASU_3139_yvnB |  | 0 | Putative exported phosphohydrolase |
| BASU_3155_ctpB |  | 0 | PDZ-containing carboxyl-terminal protease processing protease |
| BASU_3156_ywoF | exp. verified | 1 | Putative pectate lyase |
| BASU_3184_yvhJ |  | 1 | Putative membrane bound transcriptional regulator |
| BASU_3194_lytC |  | 1 | N-acetylmuramoyl-L-alanine amidase (major autolysin) |
| BASU_3195_lytB |  | 0 | Modifier protein of major autolysin lytc |
| BASU_3208_lytD | exp. verified | 0 | Exported N-acetylglucosaminidase (major autolysin) (CWBP90) |
| BASU_3215_ywtF | exp. verified | 1 | Putative transcriptional regulator |
| BASU_3218_pgdS |  | 0 | Gamma-DD-glutamyl hydrolase (PGA depolymerase) |
| BASU_3233_ywsB |  | 1 | Putative cell wall-binding protein |
| BASU_3277_ywoD |  | 14 | Putative efflux transporter |
| BASU_3290_xynA | exp. verified | 0 | Endo-14-beta-xylanase |
| BASU_3301_ywmD |  | 0 | Putative exported protein |
| BASU_3303_ywmC |  | 1 | Putative exported protein |
| BASU_3304_spoIID |  | 1 | Autolysin required for complete dissolution of the asymmetric septum (stage II sporulation) |
| BASU_3306_ywmB |  | 1 | Uncharacterized protein ywmb |
| BASU_3325_spoIIR |  | 0 | Pro-sigma(E) endopeptidase (stage II sporulation) |
| BASU_3327_ywkF |  | 3 | Uncharacterized protein ywkf |
| BASU_3415_yweA |  | 1 | Member of the processed secretome |
| BASU_3428_ywdK |  | 4 | Uncharacterized membrane protein ywdk |
| BASU_3441_vpr | exp. verified | 0 | Extracellular serine protease |
| BASU_3473_epr | exp. verified | 0 | Extracellular serine protease |
| BASU_3486_licB |  | 0 | Phosphotransferase system (PTS) lichenan-specific enzyme IIB component |
| BASU_3495_gmuB |  | 0 | Oligo-alpha-mannoside phosphotransferase system enzyme IIB |
| BASU_3501_gmuG | exp. verified | 1 | Exported mannan endo-14-beta-mannosidase |
| BASU_3523 |  | 0 | Conserved hypothetical protein |
| BASU_3527_bglS | exp. verified | 1 | Endo-beta-13-14 glucanase |
| BASU_3548_pelB |  | 1 | Pectin lyase |
| BASU_3555_abnB |  | 0 | Arabinan endo-15-alpha-L-arabinosidase |
| BASU_3600_yxaL |  | 0 | Membrane associated protein kinase with beta-propeller domain |
| BASU_3601_yxaJ |  | 4 | Putative integral membrane protein |
| BASU_3617 | unique | 3 | Conserved hypothetical protein |
| BASU_3625_yyzG |  | 2 | Uncharacterized membrane protein yyzg |
| BASU_3627_yycO |  | 0 | Uncharacterized protein yyco |
| BASU_3656_yycA |  | 0 | Conserved hypothetical protein |
| BASU_3670_sacB |  | 0 | Levansucrase |

**Genes predicted to contain Signal Peptidase II cleavage sites (SPII) Lipoproteins**

| BASU_0161_gerD |  | 0 | Lipoprotein with a role in spores' rapid response to nutrient germinants |
| --- | --- | --- | --- |
| BASU_0177_feuA | exp. verified | 0 | Iron hydroxamate-binding lipoprotein |
| BASU_0215 |  | 1 | Conserved protein of unknown function |
| BASU_0257_ansZ |  | 0 | L-asparaginase 2 (Putative lipoprotein) |
| BASU_0261_ycdA |  | 0 | Uncharacterized lipoprotein ycdA |
| BASU_0264_cwlK |  | 1 | Peptidoglycan L-alanyl-D-glutamate endopeptidase |
| BASU_0268_znuA | exp. verified | 0 | Zn(II)-binding lipoprotein |
| BASU_0281_opuAC | exp. verified | 0 | Glycine betaine ABC transporter (glycine betaine-binding lipoprotein) |
| BASU_0335_tcyA |  | 0 | Cysteine ABC transporter (substrate-binding lipoprotein) |
| BASU_0346_gerKC |  | 0 | Spore germination receptor subunit |
| BASU_0350_yxeM |  | 0 | Putative ABC transporter (binding lipoprotein) |
| BASU_0366_yclQ | exp. verified | 0 | Putative iron-siderophore ABC transporter (binding lipoprotein) |
| BASU_0396_pbpC | exp. verified | 0 | Penicillin-binding lipoprotein 3 |
| BASU_0408_ydaJ |  | 0 | Putative glycosyl hydrolase lipoprotein |
| BASU_0457_ydcC |  | 0 | Putative lipoprotein |
| BASU_0567_ydhK |  | 0 | Uncharacterized protein ydhK |
| BASU_06048_yerB |  | 0 | Putative lipoprotein |
| BASU_0654_yerH |  | 0 | Putative lipoprotein |
| BASU_0812 |  | 0 | Conserved exported protein of unknown function |
| BASU_0842_oppA1 | unique | 0 | Periplasmic oligopeptide-binding protein Flags (OppA1) |
| BASU_0850_ssuA |  | 0 | Aliphatic sulfonate ABC transporter (binding lipoprotein) |
| BASU_0877_yhcJ |  | 0 | Putative ABC transporter (binding lipoprotein) |
| BASU_0881_yhcN |  | 0 | Putative sporulation lipoprotein |
| BASU_0885_srtA |  | 1 | Sortase A |
| BASU_0957_prsA |  | 0 | Molecular chaperone lipoprotein |
| BASU_0990_yhfQ |  | 0 | Putative iron(III) dicitrate-binding lipoprotein |
| BASU_1003_yrpD | exp. verified | 0 | Uncharacterized protein yrpD |
| BASU_1004_yhjA |  | 1 | Putative Excalibur calcium-binding domain protein (lipoprotein) |
| BASU_1044_bmpA |  | 0 | Putative Basic membrane protein A (Immunodominant antigen P39) |
| BASU_1049_ipi |  | 0 | Ipi |
| BASU_1069_med |  | 0 | Positive regulator of comK |
| BASU_1077_appA |  | 0 | Oligopeptide-binding protein AppA |
| BASU_1082_oppA | exp. verified | 1 | Oligopeptide ABC transporter (binding lipoprotein) |
| BASU_1160_yjgB |  | 0 | Putative lipoprotein |
| BASU_1237_dppE |  | 0 | Dipeptide ABC transporter (dipeptide-binding lipoprotein) |
| BASU_1394_ykyA |  | 0 | Putative chromosome partitioning protein |
| BASU_1408_slp |  | 1 | Small peptidoglycan-associated lipoprotein |
| BASU_1409 |  | 0 | Putative polysaccharide deacetylase |
| BASU_1422_ylaJ |  | 0 | Putative lipoprotein |
| BASU_1432_ctaC |  | 3 | Cytochrome caa3 oxidase (subunit II) |
| BASU_1670_ymaC |  | 0 | Putative phage-related replication protein |
| BASU_1678_ymzC |  | 1 | Hypotheticalprotein |
| BASU_1722_yneA |  | 1 | Cell division inhibitor |
| BASU_1866_sodC |  | 0 | Superoxide dismutase (exported lipoprotein) |
| BASU_1892_yodJ | exp. verified | 1 | D-alanyl-D-alanine carboxypeptidase lipoprotein |
| BASU_1920_ypmR |  | 0 | Putative exported lipase/acylhydrolase (lipoprotein) |
| BASU_1921_scuA |  | 0 | Assembly factor BSco of the Cu(A) site of cytochrome c oxidase |
| BASU_2025_yphF |  | 0 | Putative lipoprotein |
| BASU_2153_artP | exp. verified | 1 | High affinity arginine ABC transporter binding lipoprotein |
| BASU_2173_yqiH |  | 0 | Uncharacterized lipoprotein yqiH |
| BASU_2233_yqhB |  | 4 | Putative membrane associated protein |
| BASU_2240_yqgU |  | 0 | Uncharacterized lipoprotein TolB C-terminal domain |
| BASU_2253_pstS | exp. verified | 0 | Phosphate ABC transporter (binding lipoprotein) |
| BASU_2312_comEA |  | 1 | Membrane bound high-affinity DNA-binding receptor |
| BASU_2325_yqeF |  | 0 | Putative lipoprotein; Putative esterase |
| BASU_2397_glnH |  | 1 | Glutamine ABC transporter (glutamine-binding lipoprotein) |
| BASU_2432_coxA |  | 1 | Spore cortex protein |
| BASU_2488_gerM |  | 0 | Germination (cortex hydrolysis) and sporulation (stage II multiple polar septa) lytic enzyme |
| BASU_2524_araN | exp. verified | 0 | Sugar-binding lipoprotein |
| BASU_2665_msmE |  | 0 | Multiple sugar-binding lipoprotein |
| BASU_2692_glpQ1 |  | 1 | Glycerophosphoryl diester phosphodiesterase (GlpQ1) |
| BASU_2709_ytkA |  | 0 | Putative exported lipoprotein |
| BASU_2785_nupN |  | 0 | Lipoprotein involved in guanosine transport |
| BASU_2834_yuiC |  | 2 | Uncharacterized protein yuiC |
| BASU_2883_frlO | exp. verified | 1 | Fructose amino acid-binding lipoprotein |
| BASU_2902_metQ |  | 1 | Methionine ABC transporter substrate binding lipoprotein |
| BASU_2925_yusW |  | 0 | Putative lipoprotein |
| BASU_2938_gerAC |  | 0 | Component of the germination receptor GerA |
| BASU_2949_yvrC | exp. verified | 1 | Putative lipoprotein binding vitamin B12 |
| BASU_2962_fhuD |  | 0 | Ferrichrome ABC transporter (ferrichrome-binding lipoprotein) |
| BASU_2968_yvgL |  | 0 | Putative molybdate-binding lipoprotein |
| BASU_2997_tcyK |  | 0 | Sulfur-containing amino acid ABC transporter binding lipoprotein |
| BASU_2998_tcyJ |  | 0 | Sulfur containing amino acid ABC transporter binding lipoprotein |
| BASU_3019_opuBC |  | 1 | Choline ABC transporter (choline-binding lipoprotein) |
| BASU_3024_opuCC |  | 1 | Glycine betaine/carnitine/choline/choline sulfate ABC transporter (osmoprotectant-binding lipoprotein) |
| BASU_3159_cccB |  | 0 | Cytochrome c551 |
| BASU_3196_lytA |  | 1 | Membrane-bound protein lytA |
| BASU_3208_lytD |  | 0 | Beta-N-acetylglucosaminidase |
| BASU_3212_gerBC |  | 0 | Lipoprotein component of the germination receptor B |
| BASU_3226_gerKC1 | exp. verified | 0 | GerKC1 |
| BASU_3232_rbsB |  | 0 | Ribose ABC transporter (ribose-binding lipoprotein) |
| BASU_3450_qoxA |  | 3 | Cytochrome aa3-600 quinol oxidase (subunit II) |
| BASU_3492 |  | 0 | Iron compound ABC transporter substrate-binding protein |
| BASU_3511_yxkH |  | 0 | Putative exported polysaccharide deacetylase lipoprotein |
| BASU_3568_yxeF |  | 0 | Uncharacterized protein yxeF |
| BASU_3572_yxeB |  | 0 | ABC transporter (ferrioxamine binding lipoprotein) |
| BASU_3713_oxaAA |  | 5 | Sec-independent factor for membrane protein insertion (YidC/SpoIIIJ family) |

**Genes predicted to contain TAT motif/cleavage sites**

| BASU_0250_phoD |  | 0 | Alkaline phosphatase D |
| --- | --- | --- | --- |
| BASU_1078_appB |  | 6 | Oligopeptide ABC transporter (permease) |
| BASU_1222_spoIISB |  | 0 | Two-component apoptotic control system component B (antitoxin) |
| BASU_1345_ykuE |  | 1 | Putative metallophosphoesterase |
| BASU_2000_qcrA |  | 1 | Menaquinol:cytochrome c oxidoreductase (iron-sulfur subunit) |
| BASU_2523_araP |  | 6 | Arabinose/arabinan permease |
| BASU_2830_bioYB |  | 6 | Putative biotin transporter |
| BASU_3401_bacH |  | 0 | Cyclohexenol-containing tetrahydro-4-hydroxyphenylpyruvate H(4)HPP in bacilysin synthesis |
| BASU_3460_efeN |  | 1 | Peroxidase converting ferric iron into ferrous iron |
| BASU_3521_yxjB |  | 0 | Putative 23S rRNA (guanine(745)-N(1))-methyltransferase |

*Experimentally verified in *B. subtilis* or *B. amyloliquefaciens* FZB42.**Table S4:** List of Transporter proteins encoded by *B. amyloliquefaciens* UCMB5113

| **Label** | **Start** | **End** | **Gene** | **Product** | **EC number** | **PubMedId** |
| --- | --- | --- | --- | --- | --- | --- |
| BASU_0061 | 65563 | 67143 | yabM | Putative exporter | _ | 15849754, 16850406 |
| BASU_0141 | 145295 | 146590 | secY | Preprotein translocase subunit | _ | 15341641, 15849754, 16850406, 9917412 |
| BASU_0150 | 151225 | 152070 | ecfA | Energizing coupling factor of ABC influx transporter (ATP-binding protein) | _ | 12110480, 21135102 |
| BASU_0151 | 152046 | 152915 | ecfAB | Energizing coupling factor of ABC influx transporter (ATP-binding protein) | _ | 12110480, 21135102 |
| BASU_0152 | 152912 | 153709 | ecfT | Component of the influx ECF transporters | _ | 15849754, 16850406, 18931129, 21135102 |
| BASU_0168 | 176934 | 178145 | glcP | Glucose/mannose transporter glcp | _ | _ |
| BASU_0172 | 181841 | 183274 | ybaR | Putative sulfate permease | _ | 15849754, 16850406 |
| BASU_0173 | 183383 | 184366 | ybaS | Putative sodium dependent transporter | _ | 15849754, 16850406 |
| BASU_0175 | 185104 | 186126 | feuC | Iron-uptake protein | _ | 15849754, 16850406, 2651410 |
| BASU_0176 | 186116 | 187123 | feuB | Iron-uptake protein | _ | 15849754, 16850406, 2651410 |
| BASU_0182 | 194442 | 195809 | murP | PTS system N-acetylmuramic acid-specific EIIBC component | 2.7.1.69 | 15060041, 15983044 |
| BASU_0192 | 206594 | 207466 | _ | Putative ATP binding protein of ABC transporter | _ | _ |
| BASU_0193 | 207444 | 208097 | _ | Putative ABC type-2 transporter | _ | _ |
| BASU_0199 | 213893 | 214390 | yxzK | Putative integral inner membrane protein involved in export murein hydrolases | _ | _ |
| BASU_0201 | 215350 | 216735 | ybxG | Putative amino acid permease | _ | 15849754, 16850406 |
| BASU_0205 | 219472 | 220407 | yfiL | Putative ABC transporter ATP-binding protein | _ | _ |
| BASU_0207 | 221600 | 222742 | yfiN1 | Yfin1, Putative ABC-type multidrug transporter | _ | _ |
| BASU_0213 | 225126 | 226763 | ybeC | Putative H+/amino acid transporter | _ | 15849754, 16850406 |
| BASU_0218 | 230644 | 231978 | glpT | Glycerol-3-phosphate permease | _ | 11929549, 15849754, 16850406, 8012593 |
| BASU_0234 | 244533 | 245783 | gltP | Proton/glutamate symport protein | _ | 15849754, 16850406, 7751298 |
| BASU_0236 | 247261 | 248763 | ybgF | Putative aminoacid permease | _ | 15849754, 16850406, 7704263 |
| BASU_0238 | 249841 | 251277 | glnT | Glutamine transporter | _ | 15849754, 15995196, 16850406 |
| BASU_0243 | 256080 | 256973 | ydfM | Putative divalent cation efflux transporter | _ | _ |
| BASU_0251 | 263080 | 263277 | tatAD | Component of the twin-arginine pre-protein translocation pathway | _ | 12867413, 15554971, 16678787, 19049517, 21479530 |
| BASU_0252 | 263338 | 264063 | tatCD | Component of the twin-arginine pre-protein translocation pathway | _ | 16698798, 19049517 |
| BASU_0255 | 265942 | 267375 | lmrB | Multidrug efflux transporter | _ | 15317768, 15849754, 16850406 |
| BASU_0269 | 282101 | 282796 | ycdI | Zn(II) transporter (ATP-binding protein) | _ | 9379902, 9811636, 21813502 |
| BASU_0270 | 282760 | 283593 | yceA | High affinity Zn(II) ABC transporter (permease) | _ | 17704766, 15849754, 16850406, 9379902, 9811636, 21813502 |
| BASU_0275 | 286760 | 287533 | yceF | Putative stress adaptation transporter | _ | 15849754, 16850406 |
| BASU_0278 | 290439 | 291641 | naiP | Niacin permease | _ | 15849754, 16850406, 18276644 |
| BASU_0279 | 292023 | 293279 | opuAA | Glycine betaine ABC transporter (ATP-binding protein) | _ | 16225868, 16645306, 7622480 |
| BASU_0280 | 293280 | 294128 | opuAB | Glycine betaine ABC transporter (permease) | _ | 15849754, 16225868, 16645306, 16850406, 8752321 |
| BASU_0287 | 301786 | 303318 | bmr3 | Multidrug-efflux transporter | _ | 15489457, 15849754, 16850406, 9023234 |
| BASU_0289 | 303957 | 304586 | ycgF | Putative aminoacid export permease | _ | 15849754, 16850406 |
| BASU_0291 | 305414 | 306742 | ycgH | Putative amino acid transporter | _ | 15849754, 16850406 |
| BASU_0296 | 309888 | 311198 | dgoT | D-galactonate transporter | _ | 12044674, 15849754, 16850406 |
| BASU_0304 | 317874 | 319328 | putP | Proline permease | _ | 14976255, 15849754, 16850406, 21840319 |
| BASU_0312 | 330994 | 332199 | nasA | Nitrate transporter | _ | 7868621, 8969502, 9384377, 19383706, 10864496, 12823818 |
| BASU_0313 | 332388 | 333590 | zinT | Zinc metallochaperone with ntpase activity | _ | 12426338, 18344368, 2013568, 9811636, 19648245 |
| BASU_0329 | 368965 | 369927 | ycxC | Putative permease | _ | 15849754, 16850406 |
| BASU_0333 | 372853 | 373596 | tcyC | Cystine ABC transporter (ATP-binding protein) | _ | 15262924 |
| BASU_0334 | 373609 | 374313 | tcyB | Cystine ABC transporter (permease) | _ | 15262924, 15849754, 16850406 |
| BASU_0335 | 374300 | 375097 | tcyA | Cystine ABC transporter (substrate-binding lipoprotein) | _ | 15262924 |
| BASU_0342 | 378983 | 380461 | dtpT | Di-tripeptide-proton ABC symporter | _ | 11717292, 15849754, 16850406, 8458848 |
| BASU_0350 | 388946 | 389737 | yxeM | Putative ABC transporter (binding lipoprotein) | _ | 12193636, 16513748, 16885442 |
| BASU_0351 | 389760 | 390434 | yxeN | Putative ABC transporter (permease) | _ | 12193636, 15849754, 16513748, 16850406, 16885442 |
| BASU_0352 | 390448 | 391197 | yxeO | Putative ABC transporter (ATP-binding protein) | _ | 12193636, 16513748, 16885442 |
| BASU_0355 | 393813 | 394496 | yclH | Putative ABC transporter (atpase component) | _ | _ |
| BASU_0356 | 394511 | 395923 | yclI | Putative transporter | _ | _ |
| BASU_0363 | 401759 | 402712 | yclN | Putative iron-siderophore ABC transporter (permease) | _ | 12354229, 15849754, 16850406 |
| BASU_0364 | 402702 | 403649 | yclO | Putative iron-siderophore ABC transporter (permease) | _ | 12354229, 15849754, 16850406 |
| BASU_0365 | 403643 | 404401 | yclP | Putative iron-siderophore ABC transporter (ATP-binding protein) | _ | 12354229 |
| BASU_0366 | 404423 | 405367 | yclQ | Putative iron-siderophore ABC transporter (binding lipoprotein) | _ | 12354229 |
| BASU_0367 | 405409 | 406833 | ycnB | Putative efflux transporter | _ | 15849754, 15855514, 16850406 |
| BASU_0375 | 413893 | 414756 | glcU | Glucose uptake protein | _ | 10438764, 15849754, 16850406 |
| BASU_0378 | 416232 | 417860 | ycnJ | Putative copper import protein | _ | 15101989, 15849754, 16850406 |
| BASU_0381 | 419168 | 420601 | mtlA | Phosphotransferase system (PTS) mannitol-specific enzyme IICB component | 2.7.1.69 | 12897001 |
| BASU_0382 | 420626 | 421057 | mtlF | Phosphotransferase system (PTS) mannitol-specific enzyme IIA component | 2.7.1.69 | 12897001 |
| BASU_0388 | 424717 | 425931 | ycsG | Putative branched chain amino acids transporter | _ | 15849754, 16850406, 9334321 |
| BASU_0413 | 452816 | 454636 | ydaO | Putative metabolite transporter | _ | 15096624, 15849754, 16850406, 20511502 |
| BASU_0428 | 462935 | 464209 | mntH | Manganese transporter | _ | 12029030, 12950915, 14580210, 15849754, 16850406 |
| BASU_0432 | 465957 | 466322 | yvaE | Putative metabolite-efflux transporter | _ | 11948170, 15849754, 16850406 |
| BASU_0441 | 473047 | 474303 | dctP | C4-dicarboxylate transport protein | _ | 10627041, 10708364, 15849754, 16850406, 20363944 |
| BASU_0443 | 475640 | 476566 | ydbJ | Putative ABC transporter (ATP-binding protein) | _ | _ |
| BASU_0444 | 476559 | 477326 | ydbK | Putative efflux ABC-transporter (permease component) | _ | 12486040, 15849754, 16850406 |
| BASU_0448 | 479570 | 480463 | ydbO | Putative cation efflux system | _ | _ |
| BASU_0485 | 510811 | 512214 | proP | Putative proline/betaine transporter | _ | _ |
| BASU_0506 | 526809 | 528101 | ybfB | Putative carboxylate transporter | _ | 15849754, 16850406 |
| BASU_0517 | 533724 | 534665 | czcD | Cadmium, cobalt and zinc/H(+)-K(+) antiporter | _ | 10735849, 12100555, 15849754, 15948947, 16850406 |
| BASU_0527 | 544372 | 545358 | _ | Putative transporter, bile acid/Na+ symporter family | _ | 12547188, 15466034 |
| BASU_0530 | 547255 | 548544 | _ | Putative Na+/H+ antiporter | _ | _ |
| BASU_0534 | 551340 | 552530 | ydeR | Putative efflux transporter | _ | 15849754, 16850406 |
| BASU_0536 | 553446 | 554330 | ydeK | Putative permease | _ | 15849754, 16850406 |
| BASU_0544 | 559464 | 561095 | vmlR | ATP-binding cassette efflux transporter | _ | 16109936 |
| BASU_0548 | 564437 | 565648 | ydgK | Putative drug resistance protein | _ | 15849754, 16850406 |
| BASU_0554 | 570430 | 571302 | _ | Eama-like transporter family protein | _ | _ |
| BASU_0568 | 580605 | 581765 | pbuE | Purine efflux pump pbue | _ | 12923093, 15849754, 16850406, 17935948 |
| BASU_0571 | 583611 | 584825 | cynX | Putative cyanate transport protein | _ | _ |
| BASU_0572 | 584885 | 585199 | yvdR | Putative membrane protein | _ | 11160890 |
| BASU_0575 | 586299 | 587861 | yveA | L-aspartate/L-glutamate permease | _ | 12730183, 15528654, 15849754, 16850406 |
| BASU_0579 | 591088 | 592506 | iolT | Myo-inositol transporter | _ | 11807058, 15849754, 16850406 |
| BASU_0586 | 602113 | 604041 | ydiF | Putative ABC transporter (ATP-binding protein) | _ | 11916677, 16109936 |
| BASU_0589 | 605356 | 605526 | tatAY | Component of the twin-arginine pre-protein translocation pathway | _ | 15187182, 19049517 |
| BASU_0590 | 605542 | 606291 | tatCY | Component of the twin-arginine pre-protein translocation pathway | _ | 11007775, 15187182, 15849754, 16850406, 19049517 |
| BASU_0603 | 622841 | 624217 | gutP | H+-glucitol symporter | _ | 12897001 |
| BASU_0619 | 638524 | 639909 | gabP | Gamma-aminobutyrate (GABA) permease | _ | 15849754, 16547045, 16850406, 8951816, 9677314 |
| BASU_0620 | 640295 | 641167 | yeaB | Putative cation efflux transporter | _ | _ |
| BASU_0629 | 652168 | 653490 | pbuG | Hypoxanthine/guanine permease | _ | 11591660, 12923093, 15849754, 16850406, 3110131 |
| BASU_0657 | 682458 | 683936 | opuE | Proline transporter | _ | 11902719, 14996799, 15849754, 16850406, 17183215, 9701821 |
| BASU_0663 | 688606 | 691749 | swrC | Swarming motility protein swrc | _ | 9384377, 11709341, 15066026 |
| BASU_0673 | 699867 | 700820 | _ | Sulfate-transporting atpase | 3.6.3.25 | _ |
| BASU_0674 | 700821 | 701696 | yfiN | Putative transport permease yfin | _ | _ |
| BASU_0706 | 728736 | 729965 | yfnC | Putative efflux transporter | _ | 15849754, 16850406 |
| BASU_0707 | 730083 | 731468 | yfnA | Metabolite permease | _ | 15849754, 16850406, 2541919 |
| BASU_0714 | 738910 | 740094 | yfmO | Metal efflux transporter | _ | 14663075, 15849754, 16850406 |
| BASU_0722 | 747159 | 748595 | yflS | 2-oxoglutarate/malate transporter | _ | 12949159, 15849754, 16850406 |
| BASU_0732 | 757754 | 759052 | citM | Transporter of divalent metal ions/citrate complexes | _ | 12427932, 12533460, 12906362, 12949159, 15849754, 16842348, 16850406 |
| BASU_0741 | 763980 | 765362 | nagP | Phosphotransferase system, N-acetylglucosamine-specific IIBC component | 2.7.1.69 | 15849754, 16850406, 8246840 |
| BASU_0748 | 769778 | 771193 | treP | Phosphotransferase system (PTS) trehalose-specific enzyme IIBC component | 2.7.1.69 | 8917076, 8969503, 9272861, 9384377, 7651129 |
| BASU_0760 | 784588 | 785781 | ydiN | Putative transporter | _ | _ |
| BASU_0766 | 788342 | 789520 | yfkF | Putative MFS-type transporter yfkf | _ | _ |
| BASU_0767 | 789679 | 790734 | yfkE | Calcium/proton exchanger | _ | _ |
| BASU_0775 | 796470 | 797429 | yfjQ | Magnesium/cobalt transport protein | _ | 10360571, 16598263, 16857941 |
| BASU_0789 | 812973 | 814559 | malP | Phosphotransferase system (PTS) maltose-specific enzyme IICB component | 2.7.1.69 | 15849754, 16707683, 16850406 |
| BASU_0790 | 814696 | 816429 | malQ | Maltose and maltodextrin ABC transporter subunit (ATP-binding protein) | _ | 16707683 |
| BASU_0791 | 816410 | 818227 | yfiC | Putative ABC transporter (ATP-binding protein) | _ | _ |
| BASU_0801 | 827043 | 828635 | yfiU | Putative efflux transporter | _ | 15849754, 16850406 |
| BASU_0811 | 836316 | 837515 | yfhI | Putative efflux transporter | _ | 15849754, 16850406 |
| BASU_0827 | 850035 | 851777 | ygaD | Putative ABC transporter (ATP-binding protein) | _ | _ |
| BASU_0836 | 864037 | 865608 | _ | Na+/H+ antiporter nhac-like protein | _ | _ |
| BASU_0842 | 870283 | 872001 | appA1 | Periplasmic oligopeptide-binding protein Flags | _ | _ |
| BASU_0843 | 872073 | 873029 | appB1 | Oligopeptide transport system permease protein | _ | _ |
| BASU_0844 | 873033 | 873947 | appC1 | Oligopeptide transport system permease protein | _ | _ |
| BASU_0845 | 873944 | 874930 | appD1 | Oligopeptide transport ATP-binding protein | _ | _ |
| BASU_0846 | 874923 | 875888 | appF1 | Oligopeptide transport ATP-binding protein (nickel importing enzyme) | _ | _ |
| BASU_0849 | 878821 | 879588 | ssuB | Aliphatic sulfonate ABC transporter (ATP-binding protein) | _ | 11390694, 16513748, 16885442, 9782504 |
| BASU_0851 | 880593 | 881426 | ssuC | Aliphatic sulfonate ABC transporter (permease) | _ | 11390694, 15849754, 16513748, 16850406, 16885442, 9782504 |
| BASU_0867 | 893986 | 894618 | yhbJ | Putative multidrug resistance efflux pump | _ | 15849754, 16850406 |
| BASU_0868 | 894659 | 896254 | yhcA | Putative exporter | _ | 15849754, 16850406, 20011599 |
| BASU_0873 | 898453 | 899169 | yhcG | Putative ABC transporter ATP-binding protein | _ | 15870467 |
| BASU_0874 | 899170 | 900087 | yhcH | Putative ABC transporter (ATP-binding protein) | _ | 15870467, 7476193 |
| BASU_0875 | 900080 | 901027 | yhcI | Putative ABC transporter (permease) | _ | 15849754, 15870467, 16850406 |
| BASU_0877 | 901702 | 902532 | yhcJ | Putative ABC transporter (binding lipoprotein) | _ | _ |
| BASU_0879 | 903780 | 905171 | tcyP | Sodium-cystine symporter | _ | 15262924, 15849754, 16850406 |
| BASU_0893 | 917645 | 918472 | glpF | Glycerol permease | _ | 11929549, 15849754, 16850406 |
| BASU_0911 | 938365 | 939768 | bcaP | Branched-chain amino acid transporter | _ | 15849754, 16621821, 16850406, 21097623 |
| BASU_0912 | 939881 | 941236 | yhdH | Putative sodium-dependent transporter | _ | 15849754, 16850406 |
| BASU_0917 | 943992 | 945326 | yhdP | Putative transporter or sensor | _ | 10960106 |
| BASU_0933 | 958140 | 959897 | yheI | ABC transporter (ATP-binding protein) involved in the signalling pathway that activates kina during sporulation initiation | _ | 15855514, 16487324 |
| BASU_0934 | 959894 | 961915 | yheH | ABC transporter (ATP-binding protein) involved in the signalling pathway that activates kina during sporulation initiation | _ | 15855514, 16487324 |
| BASU_0946 | 973266 | 974480 | yhaU | Transporter involved in K+ efflux | _ | 14987767, 15849754, 15919996, 16850406 |
| BASU_0947 | 974488 | 974985 | khtT | K+/H+ antiporter for K+ efflux | _ | 14987767, 17679694 |
| BASU_0948 | 975013 | 975366 | khtS | Putative K+/H+ antiporter for K+ efflux | _ | 14987767, 17679694 |
| BASU_0951 | 976634 | 977530 | yhaQ | Na+-efflux ABC transporter (ATP-binding protein) | _ | 10356997, 9106203 |
| BASU_0952 | 977523 | 978782 | yhaP | Na+ exporter (ABC permease) | _ | 10356997, 15849754, 16850406, 9106203 |
| BASU_0964 | 988630 | 989148 | trpP | Tryptophan transporter | _ | 14702295, 15849754, 16850406, 10735881 |
| BASU_0967 | 991412 | 992155 | ecsA | ABC transporter (ATP-binding protein) | _ | 10027970, 11807061, 8581172, 18599827 |
| BASU_0968 | 992148 | 993374 | ecsB | ABC transporter (membrane protein) | _ | 10027970, 15175311, 15849754, 16850406, 8581172, 18599827 |
| BASU_0971 | 995400 | 996785 | yhfA | Putative transporter | _ | 15849754, 16850406 |
| BASU_0982 | 1009055 | 1010344 | gltT | Proton/sodium-glutamate symport protein | _ | 15849754, 16850406, 8634258 |
| BASU_0990 | 1018541 | 1019419 | yhfQ | Putative iron(III) dicitrate-binding lipoprotein | _ | 12354229 |
| BASU_0994 | 1022588 | 1023145 | bioY | Biotin transporter | _ | 12368242, 15849754, 16850406 |
| BASU_1006 | 1032248 | 1033717 | yhjB | Putative Na+/metabolite cotransporter | _ | 15849754, 16267290, 16850406 |
| BASU_1014 | 1039283 | 1040479 | blt | Multidrug resistance protein (major facilitator transporter) | _ | 10200972, 10359661, 12793527, 15849754, 16850406, 9083003 |
| BASU_1034 | 1062557 | 1063954 | yisQ | Putative Na+driven efflux transporter | _ | 15849754, 16850406 |
| BASU_1037 | 1065603 | 1066211 | yisU | Putative aminoacid related metabolite efflux transporter | _ | 15849754, 16850406 |
| BASU_1057 | 1084332 | 1084874 | yitZ | Putative transport protein | _ | 15849754, 16850406 |
| BASU_1075 | 1101461 | 1102447 | appD | Oligopeptide ABC transporter (ATP-binding protein) | 3.6.3.- | 7997159 |
| BASU_1076 | 1102444 | 1103433 | appF | Oligopeptide ABC transporter (ATP-binding protein) | 3.6.3.- | 7997159 |
| BASU_1078 | 1105206 | 1106156 | appB | Oligopeptide ABC transporter (permease) | _ | 17704766, 15849754, 16850406, 7997159 |
| BASU_1079 | 1106178 | 1107092 | appC | Oligopeptide ABC transporter (permease) | _ | 15849754, 16850406, 7997159 |
| BASU_1082 | 1109761 | 1111395 | oppA | Oligopeptide ABC transporter (binding lipoprotein) | _ | 11083832, 11401703, 12823818, 1901616 |
| BASU_1083 | 1111502 | 1112437 | oppB | Oligopeptide ABC transporter (permease) | _ | 11401703, 15849754, 16850406, 1901616 |
| BASU_1084 | 1112441 | 1113358 | oppC | Oligopeptide ABC transporter (permease) | _ | 11401703, 15849754, 16850406, 1901616 |
| BASU_1085 | 1113371 | 1114447 | oppD | Oligopeptide ABC transporter (ATP-binding protein) | _ | 11401703, 1901616 |
| BASU_1086 | 1114440 | 1115357 | oppF | Oligopeptide ABC transporter (ATP-binding protein) | _ | 11401703, 1901616 |
| BASU_1090 | 1117979 | 1118635 | yjbE | Putative transporter component | _ | 15849754, 16850406 |
| BASU_1104 | 1130107 | 1131951 | yjbQ | Putative Na+/H+ antiporter | _ | 15849754, 16850406 |
| BASU_1126 | 1146907 | 1147629 | yvfS | Putative ABC-type multidrug transport system, permease | _ | _ |
| BASU_1127 | 1147622 | 1148359 | _ | ABC transporter ATP-binding protein | 3.6.3.- | _ |
| BASU_1144 | 1161530 | 1162942 | yfjF | Yfjf | _ | _ |
| BASU_1150 | 1168690 | 1170387 | lacE | PTS system lactose-specific EIICB component | 2.7.1.69 | 1400164, 8277252, 12397186 |
| BASU_1166 | 1184940 | 1185701 | yjkA | Putative ABC transporter (permease) | _ | 15849754, 16850406 |
| BASU_1167 | 1185703 | 1186440 | yjkB | Putative phosphate ABC transporter (ATP-binding protein) | 3.6.3.27 | _ |
| BASU_1168 | 1186467 | 1187450 | yjlA | Putative permease | _ | 15849754, 16850406 |
| BASU_1173 | 1191708 | 1193093 | exuM | Putative Na+:altronate/mannonate symporter | _ | 15849754, 16850406, 9882655 |
| BASU_1224 | 1231667 | 1232665 | pit | Low-affinity inorganic phosphate transporter | _ | 15849754, 16850406 |
| BASU_1226 | 1233581 | 1234897 | ykbA | Putative amino acid permease | _ | 15849754, 16850406 |
| BASU_1232 | 1241485 | 1243044 | yubD | Putative efflux transporter | _ | 15849754, 16850406 |
| BASU_1234 | 1244039 | 1244965 | dppB | Dipeptide ABC transporter (permease) | _ | 15849754, 16850406, 1766370, 7536291 |
| BASU_1235 | 1244962 | 1245918 | dppC | Dipeptide ABC transporter (permease) | _ | 15849754, 16850406, 1766370, 7536291 |
| BASU_1236 | 1245924 | 1246952 | dppD | Dipeptide ABC transporter (ATP-binding protein) | _ | 7536291 |
| BASU_1237 | 1246949 | 1248568 | dppE | Dipeptide ABC transporter (dipeptide-binding lipoprotein) | _ | 11390694, 7783641, 8793880 |
| BASU_1241 | 1251595 | 1252566 | ykfD | Putative cell wall oligopeptide ABC transporter (ATP binding protein) | _ | 15101989 |
| BASU_1247 | 1256116 | 1256454 | ykkC | Multidrug resistance protein ykkc | _ | 8682784, 9384377, 10735877 |
| BASU_1248 | 1256454 | 1256768 | ykkD | Efflux transporter | _ | 10735877, 10931887, 15096624 |
| BASU_1259 | 1265754 | 1266938 | hcaT | Putative 3-phenylpropionic acid transporter | _ | 9603882, 9205837, 9278503, 16738553 |
| BASU_1264 | 1272464 | 1273225 | thiX | Thiamine transporter, permease component | _ | 15849754, 16291685, 16356850, 16850406 |
| BASU_1265 | 1273200 | 1274837 | thiW | Thiamine ABC transporter (ATP-binding protein) | _ | 16291685, 16356850 |
| BASU_1266 | 1274824 | 1275423 | thiV | Thiamine transporter, permease component | _ | 15849754, 16291685, 16356850, 16850406 |
| BASU_1269 | 1277863 | 1279218 | mgtE | Magnesium transporter | _ | 15096624 |
| BASU_1284 | 1290909 | 1292261 | ktrD | K+-transporting atpase | 3.6.3.12 | 12562800, 15096624, 15849754, 16850406 |
| BASU_1305 | 1312614 | 1313651 | ykvI | Putative transporter | _ | 15383836, 15849754, 16850406 |
| BASU_1306 | 1313749 | 1314870 | _ | Conserved membrane protein of unknown function | _ | _ |
| BASU_1324 | 1326837 | 1328750 | zosA | Zinc-transporting atpase | 3.6.3.5,  3.6.3.3 | 12029044, 12180919, 15802251, 21813502 |
| BASU_1328 | 1332354 | 1334450 | ptsG | Phosphotransferase system (PTS) glucose-specific enzyme IICBA component | 2.7.1.69 | 1447219, 14527945, 15849754, 16850406, 8418852, 8432747, 9593197 |
| BASU_1329 | 1334547 | 1334813 | ptsH | Phosphocarrier protein hpr of the phosphotransferase system (PTS) | _ | 10217795, 12411438, 14527945, 15126459, 16267306, 17085448, 17142398 |
| BASU_1330 | 1334813 | 1336528 | ptsI | Phosphotransferase system (PTS) enzyme I | 2.7.3.9 | 11902727, 15155854, 15670601, 3106335 |
| BASU_1343 | 1349586 | 1350878 | ykuC | Putative efflux transporter | _ | 15849754, 16850406 |
| BASU_1360 | 1362862 | 1363668 | ykuT | Putative small-conductance mechanosensitive channel | _ | 12626684 |
| BASU_1370 | 1371113 | 1371811 | YknW | Putative permease | _ | 15849754, 16850406 |
| BASU_1371 | 1371813 | 1372949 | yknX | Putative efflux permease | _ | 11544226 |
| BASU_1372 | 1372950 | 1373642 | yknY | Putative ABC transporter (ATP-binding protein) | 3.6.3.28 | _ |
| BASU_1373 | 1373639 | 1374832 | yknZ | Putative permease | _ | _ |
| BASU_1376 | 1376792 | 1378696 | fruA | Phosphotransferase system (PTS) fructose-specific enzyme IIABC component | 2.7.1.69 | 118007, 200418 |
| BASU_1378 | 1379446 | 1379715 | ykoA | Conserved hypothetical protein | _ | _ |
| BASU_1379 | 1379875 | 1381494 | ykpA | ABC efflux transporter (ATP-binding protein) | _ | 10816464 |
| BASU_1387 | 1387995 | 1388660 | ktrC | Potassium uptake protein | _ | 12562800, 15096624 |
| BASU_1427 | 1471073 | 1472284 | ftsW | Cell-division protein | _ | 12626683, 15849754, 16850406, 9622350, 21386816 |
| BASU_1473 | 1522773 | 1523570 | ylmA | Putative ABC transporter (ATP-binding protein) | _ | 16707683 |
| BASU_1487 | 1535195 | 1536505 | pyrP | Uracil permease | _ | 15849754, 16850406, 8206848 |
| BASU_1497 | 1547190 | 1548254 | cysP | Sulfate permease | _ | 10784039, 15849754, 16850406, 11004190, 10094622 |
| BASU_1504 | 1554484 | 1557156 | yloB | P-type calcium transport atpase | _ | 12161109 |
| BASU_1575 | 1621421 | 1622086 | fliP | Component of the flagellar export machinery | _ | 15516571, 15849754, 16850406 |
| BASU_1576 | 1622092 | 1622370 | fliQ | Component of the flagellar export machinery | _ | 10234819, 15516571 |
| BASU_1577 | 1622377 | 1623156 | fliR | Component of the flagellar export machinery | _ | 15060055, 15516571, 15849754, 16850406 |
| BASU_1578 | 1623153 | 1624235 | flhB | Component of the flagellar export machinery | _ | 10234819, 16949608, 17050924, 17067800 |
| BASU_1580 | 1626302 | 1627393 | flhF | Gtpase involved in the export of flagella | _ | 1447978, 15317803, 16980502, 17565194, 17699634 |
| BASU_1623 | 1671887 | 1673215 | ymfD | Bacillibactin exporter | _ | 15849754, 16850406, 18502870 |
| BASU_1625 | 1673777 | 1676896 | _ | Putative integral membrane protein | _ | _ |
| BASU_1672 | 1781833 | 1782192 | ebrB | Small multidrug efflux transporter | _ | 10735876, 15849754, 16750162, 16850406 |
| BASU_1673 | 1782204 | 1782536 | ebrA | Small multidrug resistance efflux transporter | _ | 10735876, 11104814, 17516673 |
| BASU_1691 | 1798369 | 1799760 | xynP | Putative H+-xyloside symporter | _ | 15849754, 16684115, 16850406, 17183216 |
| BASU_1711 | 1821473 | 1822093 | chbA | Putative chitin binding protein | _ | _ |
| BASU_1750 | 1852330 | 1853748 | alsT | Amino acid carrier protein | _ | 12823818, 15849754, 16850406, 8969507 |
| BASU_1757 | 1859678 | 1860949 | exuT | Hexuronate transporter | _ | 15849754, 16850406, 8320243, 9579062, 9882655 |
| BASU_1761 | 1864361 | 1864570 | _ | Putative MFS-type transporter | _ | _ |
| BASU_1778 | 1914127 | 1915563 | yxjC | Putative permease | _ | 15849754, 16850406 |
| BASU_1804 | 1974987 | 1976375 | yoeA | Putative efflux transporter | _ | 15849754, 16850406 |
| BASU_1809 | 1980564 | 1981739 | ybcL | Putative efflux transporter | _ | 15812018, 15849754, 16850406 |
| BASU_1823 | 1996802 | 1998049 | yoaB | Negatively charged metabolite transporter | _ | 12193636, 15849754, 16513748, 16850406 |
| BASU_1859 | 2027716 | 2029053 | yocR | Putative sodium-dependent transporter | _ | 15849754, 16850406 |
| BASU_1860 | 2029257 | 2030231 | yocS | Putative sodium-dependent transporter | _ | 15849754, 16850406 |
| BASU_1871 | 2042777 | 2044138 | norM | Mate Na+-driven efflux family protein | _ | 14766544, 15849754, 15855508, 16850406, 16954325 |
| BASU_1885 | 2052226 | 2053716 | yodF | Putative Na+/metabolite permease | _ | 12823818, 15849754, 16850406 |
| BASU_1956 | 2112971 | 2114275 | pbuX | Xanthine permease | _ | 12923093, 15549109, 15849754, 16850406 |
| BASU_1967 | 2126243 | 2126749 | crr | Glucose-specific phosphotransferase enzyme IIA component | 2.7.1.69 | 1606145, 1630305, 1911770, 1961703, 2014267, 2457575, 2960675, 8170944, 8430315, 8764513, 9298646, 9405042, 9600841 |
| BASU_2048 | 2199550 | 2200122 | fmnP | FMN permease | _ | 12456892, 15849754, 16850406, 17693491 |
| BASU_2096 | 2240244 | 2241650 | mleN | Malate-H+/Na+-lactate antiporter | _ | 10903309, 15849754, 16850406 |
| BASU_2151 | 2347282 | 2348004 | artR | High affinity arginine ABC transporter (ATP-binding protein) | _ | 10939241, 11423008 |
| BASU_2152 | 2347997 | 2348656 | artQ | High affinity arginine ABC transporter (permease) | _ | 10939241, 11423008, 15849754, 16850406 |
| BASU_2153 | 2348718 | 2349485 | artP | High affinity arginine ABC transporter binding lipoprotein | _ | 10939241, 11423008 |
| BASU_2232 | 2417533 | 2418483 | yqxL | Putative cora-type Mg(2+) transporter | _ | 15856219 |
| BASU_2244 | 2426950 | 2428314 | nhaC | Na+/H+ antiporter | _ | 10735849, 11274110, 15849754, 16850406 |
| BASU_2249 | 2430677 | 2431459 | pstBB | Phosphate ABC transporter (ATP-binding protein) | _ | 12897025, 15289558, 9098050 |
| BASU_2250 | 2431471 | 2432277 | pstBA | Phosphate ABC transporter (ATP-binding protein) | _ | 12897025, 15289558, 9098050 |
| BASU_2251 | 2432296 | 2433180 | pstA | Phosphate ABC transporter (permease) | _ | 12897025, 15289558, 15849754, 16850406, 9098050 |
| BASU_2252 | 2433180 | 2434109 | pstC | Phosphate ABC transporter (permease) | _ | 12897025, 15289558, 15849754, 16850406, 9098050 |
| BASU_2253 | 2434158 | 2435060 | pstS | Phosphate ABC transporter (binding lipoprotein) | _ | 10913081, 15289558, 9098050, 9593301 |
| BASU_2255 | 2437465 | 2438754 | yqgE | Putative efflux transporter | _ | 10960106, 15849754, 16850406 |
| BASU_2295 | 2472985 | 2473920 | yqeW | Putative Na+/anion cotransporter | _ | 15849754, 16850406 |
| BASU_2329 | 2503002 | 2504258 | _ | Uncharacterized MFS-type transporter | _ | _ |
| BASU_2335 | 2511503 | 2512951 | mdtD | Putative multidrug resistance protein mdtd | _ | 7916693, 8440253, 1328813, 8577249 |
| BASU_2355 | 2539386 | 2540762 | tetB | Tetracycline resistance protein | _ | 14636065, 15849754, 16850406, 17015648, 7961647 |
| BASU_2363 | 2546542 | 2548500 | manP | Phosphotransferase system (PTS) mannose-specific enzyme IIBCA component | 2.7.1.69 | 10627040, 10960106 |
| BASU_2368 | 2553355 | 2554563 | yybF | Uncharacterized MFS-type transporter | _ | 15849754, 16850406, 9811656 |
| BASU_2373 | 2560255 | 2561055 | yrhG | Putative formate/nitrite transporter | _ | 15849754, 16850406 |
| BASU_2395 | 2581925 | 2582986 | yrrI | Putative permease | _ | 15849754, 16850406 |
| BASU_2396 | 2583207 | 2583935 | glnQ | Glutamine ABC transporter (ATP-binding protein) | 3.6.3.- | 1856180 |
| BASU_2397 | 2583956 | 2584783 | glnH | Glutamine ABC transporter (glutamine-binding lipoprotein) | _ | 1856180 |
| BASU_2398 | 2584840 | 2585490 | glnM | Glutamine ABC transporter (permease) | _ | 15849754, 16850406, 1856180 |
| BASU_2399 | 2585509 | 2586165 | glnP | Glutamine ABC transporter (permease) | _ | 15849754, 16850406, 1856180 |
| BASU_2418 | 2607092 | 2607589 | yrvC | Putative potassium transport accessory component | _ | 14563871 |
| BASU_2422 | 2607721 | 2609940 | secDF | Protein-export membrane protein | _ | 15941986, 15995216, 9694879 |
| BASU_2419 | 2610389 | 2611945 | spoVB | Putative Putative translocase with flippase function for teichoic acid synthesis;  Involved in spore cortex synthesis (stage V sporulation) | _ | 15292147, 15849754, 16850406, 1744050 |
| BASU_2423 | 2612776 | 2613162 | yrzE | Putative transporter | _ | 15849754, 16850406 |
| BASU_2424 | 2613214 | 2613474 | yrbF | Component of the preprotein translocase | _ | 10974125, 16166550, 21235483 |
| BASU_2522 | 2713619 | 2714461 | araQ | Arabinose/arabinan permease | _ | 10417639, 11418559, 14973026, 15849754, 16850406 |
| BASU_2523 | 2714465 | 2715406 | araP | Arabinose/arabinan permease | _ | 10417639, 11418559, 14973026, 15849754, 16850406 |
| BASU_2551 | 2741699 | 2742898 | ytbD | Putative transporter | _ | 15849754, 16850406 |
| BASU_2568 | 2759709 | 2760824 | ytvI | Putative permease | _ | 12662922, 15849754, 16850406 |
| BASU_2600 | 2793625 | 2794965 | braB | Branched-chain amino acid-Na+ symporter | _ | 11528000, 15849754, 16850406 |
| BASU_2617 | 2811477 | 2812346 | _ | Cation efflux protein | _ | _ |
| BASU_2619 | 2813448 | 2814179 | _ | Putative amino acid permease (azlc-like protein) | _ | _ |
| BASU_2640 | 2833226 | 2833774 | _ | HPP family protein | _ | _ |
| BASU_2654 | 2846540 | 2847844 | pbuO | Hypoxanthine/guanine permease | _ | 11591660, 15849754, 16850406 |
| BASU_2655 | 2847878 | 2849038 | ythQ | Putative ABC transporter (permease) | _ | _ |
| BASU_2656 | 2849035 | 2849739 | ythP | Putative ABC transporter (ATP-binding protein) | 3.6.3.- | _ |
| BASU_2662 | 2854435 | 2855970 | opuD | Glycine betaine transporter | _ | 14996799, 15849754, 16850406, 8752321, 9925583 |
| BASU_2666 | 2858730 | 2859644 | msmF | Multiple sugar-binding transport system permease protein msmf | _ | 12847288, 1537846, 15849754, 16707683, 16850406 |
| BASU_2667 | 2859641 | 2860462 | msmG | Multiple sugar-binding transport system permease protein msmg | _ | 1537846, 15849754, 16707683, 16850406 |
| BASU_2674 | 2867185 | 2868369 | yttB | Putative efflux transporter | _ | 11988534, 15849754, 16850406 |
| BASU_2677 | 2869392 | 2871332 | bceB | ABC transporter (permease) | _ | 14612242, 15849754, 16850406, 17905982 |
| BASU_2678 | 2871329 | 2872084 | bceA | Bacitracin ABC efflux transporter (ATP-binding protein) | _ | 14612242, 17905982 |
| BASU_2681 | 2873943 | 2875253 | ytrF | Metabolite permease | _ | 10986249, 15849754, 16850406 |
| BASU_2682 | 2875243 | 2875947 | ytrE | ABC transporter (ATP-binding protein) | _ | 10986249 |
| BASU_2683 | 2875954 | 2876958 | ytrD | Putative ABC transporter permease | _ | _ |
| BASU_2684 | 2876971 | 2877957 | ytrC1 | Similar to ABC transporter permease | _ | 10986249, 15849754, 16850406 |
| BASU_2685 | 2877973 | 2878959 | ytrC | ABC transporter permease ytrc | _ | 10986249, 15849754, 16850406 |
| BASU_2686 | 2878953 | 2879831 | ytrB | ABC transporter (ATP-binding protein) | 3.6.3.- | 10986249 |
| BASU_2696 | 2886493 | 2887896 | ytnA | Putative amino acid permease | _ | 10498721, 15849754, 16850406 |
| BASU_2702 | 2894688 | 2895692 | ytlA | Putative ABC transporter component | _ | _ |
| BASU_2703 | 2895704 | 2896486 | ytlC | Putative ABC transporter component, ATP-binding | _ | _ |
| BASU_2704 | 2896461 | 2897273 | ytlD | Putative permease of ABC transporter | _ | 15849754, 16850406 |
| BASU_2726 | 2921341 | 2921922 | thiT | Thiamin permease | _ | 15849754, 16291685, 16356850, 16850406 |
| BASU_2736 | 2929636 | 2930304 | ktrA | Potassium uptake protein | _ | 12562800, 15096624 |
| BASU_2755 | 2950808 | 2952097 | yugS | Putative membrane protein involved in divalent ion export | _ | _ |
| BASU_2759 | 2953510 | 2954496 | yugO | Putative potassium channel protein | _ | 16704729 |
| BASU_2778 | 2969049 | 2970293 | yuxJ | Putative exporter | _ | 15849754, 16850406 |
| BASU_2786 | 2977357 | 2978889 | nupO | Guanosine ABC transporter (ATP-binding protein) | _ | 21926227 |
| BASU_2787 | 2978882 | 2979928 | nupP | Permease of ABC guanosine transporter | _ | 15849754, 16850406, 21926227 |
| BASU_2788 | 2979929 | 2980888 | nupQ | Permease of ABC guanosine transporter | _ | 15849754, 16850406, 21926227 |
| BASU_2789 | 2981396 | 2983798 | mrpA | Sodium transporter component of a Na+/H+ antiporter | _ | 9274030, 9384377, 9878723, 10198001, 10648512, 11356194, 17293423 |
| BASU_2790 | 2983795 | 2984226 | mrpB | Na+/H+ antiporter complex | _ | 9274030, 9384377, 10198001, 11356194, 17293423 |
| BASU_2791 | 2984226 | 2984567 | mrpC | Component of Na+/H+ antiporter | _ | 11004162, 11356194, 12682299, 15019740, 15849754, 16850406, 17693497 |
| BASU_2792 | 2984551 | 2986041 | mrpD | Proton transporter component of Na+/H+ antiporter | _ | 11004162, 11356194, 12682299, 15019740, 15849754, 16850406, 21236240 |
| BASU_2793 | 2986047 | 2986523 | mrpE | Non essential component of Na+/H+ antiporter | _ | 15849754, 16233411, 16850406, 11004162, 10198001 |
| BASU_2794 | 2986523 | 2986807 | mrpF | Efflux transporter for Na+ and cholate | _ | 10198001, 11004162, 11356194, 12682299, 15019740 |
| BASU_2795 | 2986791 | 2987165 | mrpG | Non essential component of Na+/H+ antiporter | _ | 11004162, 11356194, 15019740, 15849754, 16850406 |
| BASU_2830 | 3028007 | 3028600 | bioYB | Putative biotin transporter | _ | 11717296, 15849754, 16850406 |
| BASU_2831 | 3028647 | 3029975 | yuiF | Amino acid transporter | _ | 15135544, 15849754, 16850406 |
| BASU_2835 | 3032994 | 3033314 | yuiB | Uncharacterized membrane protein yuib | _ | _ |
| BASU_2842 | 3038415 | 3039119 | _ | Putative ABC-transporter ATP-binding protein | _ | _ |
| BASU_2850 | 3045321 | 3046538 | yutK | Putative Na+(H+)/nucleoside cotransporter | _ | 15849754, 16850406 |
| BASU_2874 | 3069826 | 3071022 | _ | Putative multidrug resistance protein | _ | 10200972, 10359661, 12793527, 15849754, 16850406, 9083003 |
| BASU_2878 | 3074776 | 3075882 | yurJ | Putative multiple sugar ABC transporter (ATP-binding protein) | _ | _ |
| BASU_2881 | 3077659 | 3078543 | frlM | Fructose-amino acid permease | _ | 12618455, 15849754, 16850406, 15556630, 21347729 |
| BASU_2882 | 3078547 | 3079437 | frlN | Fructose-amino acid permease | _ | 12618455, 15849754, 16850406, 15556630, 21347729 |
| BASU_2883 | 3079462 | 3080748 | frlO | Fructose amino acid-binding lipoprotein | _ | 12618455, 21347729 |
| BASU_2902 | 3095456 | 3096271 | metQ | Methionine ABC transporter, substrate binding lipoprotein | _ | 12910260, 14990259, 17038623, 10094622 |
| BASU_2903 | 3096285 | 3096953 | metP | Methionine ABC transporter, permease component | _ | 12910260, 14990259, 15849754, 16850406, 17038623, 10094622 |
| BASU_2904 | 3096946 | 3097971 | metN | Methionine ABC transporter (ATP-binding protein) | _ | 12910260, 14990259, 17038623, 10094622 |
| BASU_2919 | 3108641 | 3110236 | yusP | Putative multidrug-efflux transporter | _ | 15849754, 16850406 |
| BASU_2924 | 3113622 | 3114446 | yusV | Iron(III)-siderophore transporter (ATP binding component) | _ | 12354229, 16672620 |
| BASU_2944 | 3133000 | 3133380 | liaI | Permease | _ | 15273097, 15849754, 16816187, 16850406 |
| BASU_2945 | 3133547 | 3134815 | yvqJ | Putative efflux protein | _ | 15849754, 16850406 |
| BASU_2947 | 3135546 | 3136772 | yvrA | Putative Phosphonate/Iron-chelate transporting atpase | 3.6.3.- | _ |
| BASU_2948 | 3136769 | 3137815 | yvrB | Putative vitamin B12 permease | _ | 14704351, 15849754, 16850406 |
| BASU_2949 | 3137778 | 3138722 | yvrC | Putative lipoprotein binding vitamin B12 | _ | 14704351 |
| BASU_2959 | 3146300 | 3147103 | fhuC | Ferrichrome ABC transporter (ATP-binding protein) | _ | 15802251, 8388528, 8596459 |
| BASU_2960 | 3147122 | 3148132 | fhuG | Ferrichrome ABC transporter (permease) | _ | 15802251, 15849754, 16850406, 8388528, 8596459 |
| BASU_2961 | 3148132 | 3149199 | fhuB | Ferrichrome ABC transporter (permease) | _ | 12354229, 15849754, 16850406, 8388528 |
| BASU_2962 | 3149417 | 3150355 | fhuD | Ferrichrome ABC transporter (ferrichrome-binding lipoprotein) | _ | 12354229, 8388528 |
| BASU_2963 | 3150530 | 3151942 | lysP | Lysine permease | _ | 14627808, 15849754, 16850406 |
| BASU_2969 | 3156912 | 3157595 | yvgM | Putative molybdenum transport permease | _ | 15849754, 16850406 |
| BASU_2972 | 3159566 | 3160501 | _ | Putative ABC transporter ATP-binding protein | _ | _ |
| BASU_2973 | 3160525 | 3161619 | _ | Putative ABC-2 type transporter | _ | _ |
| BASU_2974 | 3161621 | 3162766 | _ | Putative ABC-2 type transporter | _ | _ |
| BASU_2977 | 3164376 | 3166391 | nhaK | Na+/H+ antiporter | _ | 15849754, 16021482, 16850406 |
| BASU_2985 | 3175816 | 3177927 | cadA | Cadmium, zinc and cobalt-transporting atpase | 3.6.3.3,  3.6.3.5 | 9384377, 11267663, 11934502, 12779235, 16901659 |
| BASU_2986 | 3178075 | 3180504 | copA | Copper-exporting P-type atpase A | 3.6.3.4 | 11922674, 11934502, 12644235, 14663075, 18215122 |
| BASU_2994 | 3185858 | 3186637 | tcyN | Sulfur-containing amino-acid ABC transporter (ATP-binding protein) | _ | 10939241, 11423008, 15262924 |
| BASU_2995 | 3186634 | 3187341 | tcyM | Sulfur-containing amino acid ABC transporter (permease) | _ | 10939241, 11423008, 15262924, 15849754, 16850406 |
| BASU_2996 | 3187357 | 3188079 | tcyL | Sulfur-containing amino acid ABC transporter (permease) | _ | 10939241, 11423008, 15262924, 15849754, 16850406 |
| BASU_2997 | 3188102 | 3188914 | tcyK | Sulfur-containing amino acid ABC transporter binding lipoprotein | _ | 10939241, 11423008, 15262924 |
| BASU_2998 | 3188940 | 3189752 | tcyJ | Sulfur containing amino acid ABC transporter binding lipoprotein | _ | 10939241, 11423008, 15262924 |
| BASU_3018 | 3205269 | 3205946 | opuBD | Glycine betaine/carnitine/choline/choline sulfate ABC transporter permease (opucd like) | _ | 10216873, 15849754, 16850406, 9925583 |
| BASU_3019 | 3205965 | 3206882 | opuBC | Choline ABC transporter (choline-binding lipoprotein) | _ | 10216873, 9925583 |
| BASU_3020 | 3206897 | 3207550 | opuBB | Glycine betaine/carnitine/choline/choline sulfate ABC transporter (permease) | _ | 10216873, 15849754, 16850406, 9925583 |
| BASU_3021 | 3207567 | 3208712 | opuBA | Choline ABC transporter (ATP-binding protein) | _ | 10216873, 9925583, 21658392 |
| BASU_3023 | 3209565 | 3210239 | opuCD | Glycine betaine/carnitine/choline/choline sulfate ABC transporter (permease) | _ | 10216873, 15849754, 16850406, 9925583 |
| BASU_3024 | 3210257 | 3211174 | opuCC | Glycine betaine/carnitine/choline/choline sulfate ABC transporter  (osmoprotectant-binding lipoprotein) | _ | 10216873, 9925583 |
| BASU_3025 | 3211188 | 3211841 | opuCB | Glycine betaine/carnitine/choline/choline sulfate ABC transporter (permease) | _ | 10216873, 15849754, 16850406, 9925583 |
| BASU_3026 | 3211862 | 3213001 | opuCA | Glycine betaine/carnitine/choline/choline sulfate ABC transporter  (ATP-binding protein) | _ | 10216873, 9925583, 21658392 |
| BASU_3029 | 3214910 | 3215620 | yvbI | Putative permease | _ | 15849754, 16850406 |
| BASU_3032 | 3218088 | 3219323 | yfiS | Putative efflux transporter | _ | 15849754, 16850406, 16862575, 8971709, 12959402 |
| BASU_3048 | 3236347 | 3237741 | araE | Arabinose-related compounds permease | _ | 15849754, 16850406, 9620981 |
| BASU_3053 | 3241822 | 3242739 | cyeB | Cysteine and O-acetylserine efflux permease | _ | 10844694, 15849754, 16850406 |
| BASU_3054 | 3242773 | 3244113 | yvbW | Putative leucine permease | _ | 15849754, 16850406, 19258532 |
| BASU_3058 | 3247417 | 3248745 | gntT | High-affinity gluconate permease (gntp family) | _ | _ |
| BASU_3064 | 3254005 | 3255681 | yvfH | Putative lactate permease | _ | 15849754, 16850406 |
| BASU_3071 | 3260709 | 3262226 | epsK | Putative extracellular matrix component exporter | _ | 15175311, 15661000, 15849754, 16850406 |
| BASU_3097 | 3284010 | 3285242 | fruP | Sugar transporter frup | _ | 12395198 |
| BASU_3102 | 3289701 | 3291077 | _ | Putative metabolite transport protein | _ | 15849754, 16850406, 9529885 |
| BASU_3105 | 3292376 | 3293965 | yvdB | Putative sulfate transporter | _ | 15849754, 16850406 |
| BASU_3121 | 3307671 | 3309440 | bmrA | Efflux transporter (ATP-binding and permease protein) | 3.6.3.44 | 16405427, 18215075 |
| BASU_3150 | 3338920 | 3340257 | yvkA | Putative efflux transporter | _ | 15849754, 16850406 |
| BASU_3157 | 3348026 | 3348943 | ftsX | Cell division ABC transporter (ATP-binding protein) | _ | 16352817, 17071757, 17307852, 18573177 |
| BASU_3158 | 3348909 | 3349595 | ftsE | Cell-division ABC transporter (ATP-binding protein) | _ | 16352817, 17071757, 17307852, 18573177 |
| BASU_3177 | 3365016 | 3365693 | comFC | Putative component of the DNA transport apparatus | _ | _ |
| BASU_3192 | 3380600 | 3382048 | tuaB | Putative exporter involved in biosynthesis of teichuronic acid | _ | 10048024, 11994144, 15849754, 16850406 |
| BASU_3200 | 3390365 | 3391909 | tagH | ATP-binding teichoic acid precursor transporter component | _ | 12682299, 17012386, 18156271, 7565096 |
| BASU_3201 | 3391930 | 3392757 | tagG | Teichoic acid precursors permease | _ | 12682299, 15849754, 16850406, 17012386, 18156271, 7565096 |
| BASU_3214 | 3408771 | 3410147 | ywtG | Putative carbohydrate transporter | _ | 15849754, 16850406 |
| BASU_3229 | 3424043 | 3424438 | rbsD | Ribose ABC transporter (membrane bound ribose binding) | _ | 7921236 |
| BASU_3230 | 3424455 | 3425948 | rbsA | Ribose ABC transporter (ATP-binding protein) | _ | 7921236 |
| BASU_3231 | 3425938 | 3426909 | rbsC | Ribose ABC transporter (permease) | _ | 15849754, 16850406, 7921236 |
| BASU_3232 | 3426923 | 3427840 | rbsB | Ribose ABC transporter (ribose-binding lipoprotein) | _ | 7921236 |
| BASU_3238 | 3432986 | 3434317 | ywrK | Putative Na+/H+ antiporter | _ | 15849754, 16850406 |
| BASU_3246 | 3441431 | 3442021 | ywrB | Putative anion transporter | _ | 15849754, 16850406 |
| BASU_3247 | 3442018 | 3442554 | ywrA | Putative anion transporter | _ | 15849754, 16850406 |
| BASU_3267 | 3459710 | 3460105 | mscL | Large conductance mechanosensitive channel protein | _ | 12948773, 15063854, 15165739, 16897034 |
| BASU_3275 | 3465900 | 3467096 | ywoG | Putative efflux transporter | _ | 15849754, 16850406 |
| BASU_3276 | 3467131 | 3468585 | pucI | Allantoin permease | _ | 11344136, 12029039, 15849754, 16850406 |
| BASU_3277 | 3468749 | 3470113 | ywoD | Putative efflux transporter | _ | 15849754, 16850406 |
| BASU_3279 | 3470929 | 3472140 | amtB | Ammonium transporter | _ | 14600241, 15849754, 16850406, 17001076, 21435182 |
| BASU_3332 | 3513210 | 3514166 | ywkB | Auxin efflux carrier family protein | _ | 18604494, 12949160, 15849754, 16850406 |
| BASU_3349 | 3531850 | 3533574 | ywjA | Putative ABC lipid transporter (ATP-binding protein) | _ | 12119303, 12354229 |
| BASU_3359 | 3544770 | 3545960 | narK | Nitrite extrusion permease | _ | 15849754, 16796679, 16850406, 8682783, 8846791 |
| BASU_3361 | 3547761 | 3548189 | ywiB | Putative Lipocalins protein | _ | _ |
| BASU_3383 | 3562908 | 3564290 | _ | Putative amino acid permease | _ | _ |
| BASU_3390 | 3568784 | 3569704 | _ | Putative ABC transporter ATP-binding protein | _ | _ |
| BASU_3391 | 3569701 | 3570654 | _ | Putative ABC-type transport system involved in multi-copper enzyme  Maturation, permease component | _ | _ |
| BASU_3403 | 3580605 | 3581786 | bacE | Efflux protein for bacilysin excretion, self-protection against bacilysin | _ | 12372825, 15609023, 15849754, 16850406 |
| BASU_3408 | 3585453 | 3586691 | ywfA | Uncharacterized MFS-type transporter ywfa | _ | 15609023, 15849754, 16850406 |
| BASU_3409 | 3586914 | 3588116 | bcr | Bicyclomycin (Sulfonamide) resistance protein | _ | 8486276, 9278503, 16738553, 2694948, 15919996 |
| BASU_3410 | 3588149 | 3589567 | rocC | Arginine/ornithine permease | _ | 15849754, 16850406, 7540694, 8113162 |
| BASU_3429 | 3606718 | 3608037 | ywdJ | Putative purine/pyrimidine permease | _ | 15849754, 16850406 |
| BASU_3439 | 3614434 | 3615816 | sacP | Phosphotransferase system (PTS) sucrose-specific enzyme IIBC component | 2.7.1.69 | 1577686, 15849754, 16850406, 3122206 |
| BASU_3461 | 3637256 | 3637450 | ywbM | Putative periplasmic lipoprotein involved in iron transport | _ | _ |
| BASU_3472 | 3645112 | 3646446 | ywbA | Phosphotransferase system sugar-specific enzyme IIC permease component | _ | 15849754, 16850406, 8990303 |
| BASU_3484 | 3658601 | 3658933 | licA | Phosphotransferase system (PTS) lichenan-specific enzyme IIA component | _ | 10438772, 8990303 |
| BASU_3485 | 3658952 | 3660310 | licC | Phosphotransferase system (PTS) lichenan-specific enzyme IIC component | _ | 10438772, 15849754, 16850406 |
| BASU_3486 | 3660326 | 3660634 | licB | Lichenan-specific phosphotransferase enzyme IIB component | 2.7.1.69 | 10438772, 8990303 |
| BASU_3490 | 3663824 | 3664852 | fhuB | Ferrichrome ABC transporter (permease) | _ | 9639930, 9384377, 8388528 |
| BASU_3491 | 3664853 | 3665866 | _ | Ferrichrome ABC transporter Permease | _ | 15802251, 15849754, 16850406, 8388528, 8596459 |
| BASU_3492 | 3665883 | 3666779 | _ | ABC-type Fe3+-hydroxamate transport system | _ | _ |
| BASU_3494 | 3667246 | 3668544 | ydfA | Arsenical pump membrane protein (arsb like) | _ | 1534328 |
| BASU_3495 | 3668697 | 3669008 | gmuB | Oligo-beta-mannoside-specific phosphotransferase enzyme IIB component | 2.7.1.69 | 18177310 |
| BASU_3496 | 3669023 | 3669340 | gmuA | Oligo-beta-mannoside-specific phosphotransferase enzyme IIA component | _ | _ |
| BASU_3497 | 3669359 | 3670678 | gmuC | Oligo-beta-mannoside-specific phosphotransferase enzyme IIC | _ | 15849754, 16850406, 18177310 |
| BASU_3504 | 3678084 | 3679442 | yxlA | Putative purine-cytosine permease | _ | 15849754, 16850406, 2191181 |
| BASU_3506 | 3680377 | 3682107 | cydD | ABC membrane transporter (ATP-binding protein) required for cytochrome bb' function | _ | 15231791, 16040611, 9852001, 10551842 |
| BASU_3507 | 3682104 | 3683807 | cydC | ABC membrane transporter (ATP-binding protein) required for cytochrome bb' function | _ | 15231791, 16040611, 9852001, 10551842 |
| BASU_3510 | 3686716 | 3688059 | cimH | Citrate/malate/H+ symporter | _ | 11566984, 15849754, 16850406 |
| BASU_3512 | 3689036 | 3690136 | msmX | Multiple sugar-binding transporter ATP-binding protein | _ | 16707683 |
| BASU_3514 | 3691314 | 3692150 | yxkD | Efflux transporter | _ | 10735877, 10931887, 15096624, 15849754, 16850406, 21317561 |
| BASU_3522 | 3699934 | 3701127 | nupG | Purine nucleoside transporter | _ | 12787499, 15849754, 16850406 |
| BASU_3526 | 3704809 | 3706089 | citH | Secondary transporter of divalent metal ions/citrate complexes | _ | 11053381, 15849754, 16850406, 8892821 |
| BASU_3529 | 3708100 | 3709347 | yxiO | Putative efflux transporter | _ | 12583894, 15849754, 16850406 |
| BASU_3549 | 3720753 | 3721664 | yxxF | Putative transporter | _ | 15849754, 16850406 |
| BASU_3552 | 3723756 | 3725591 | bglP | Phosphotransferase system (PTS) beta-glucoside-specific enzyme IIBCA  Component | 2.7.1.69 | 15849754, 16850406, 17074746, 8626332 |
| BASU_3561 | 3737617 | 3739035 | hutM | Histidine permease | _ | 15849754, 16850406, 7704263 |
| BASU_3563 | 3740404 | 3741585 | nupC | Pyrimidine-nucleoside Na+(H+) cotransporter | _ | 11065368, 15849754, 16850406, 8550462 |
| BASU_3572 | 3746848 | 3747810 | yxeB | ABC transporter (ferrioxamine binding lipoprotein) | _ | 12354229, 16672620, 8388528 |
| BASU_3574 | 3748212 | 3750080 | yxdM | ABC transporter (permease); efflux of cationic peptides | _ | 15289557, 15849754, 15870467, 16850406 |
| BASU_3575 | 3750055 | 3750828 | yxdL | ABC transporter (ATP-binding protein); efflux of cationic peptides | _ | 15289557, 15870467 |
| BASU_3582 | 3756494 | 3757789 | iolF | Inositol transport protein | _ | 11807058, 15849754, 16850406 |
| BASU_3590 | 3766350 | 3767738 | csbC | Putative sugar transporter | _ | 10376822, 15849754, 16850406 |
| BASU_3603 | 3779211 | 3780377 | _ | Putative transporter | _ | _ |
| BASU_3609 | 3785448 | 3786794 | ydgF | D-serine/D-alanine/glycine transporter | _ | _ |
| BASU_3632 | 3808820 | 3810220 | rocE | Arginine/ornithine/gamma-aminobutyrate permease | _ | 10648515, 15849754, 16850406, 7540694 |
| BASU_3639 | 3815163 | 3816788 | _ | Putative ABC transporter ATP-binding protein | _ | _ |
| BASU_3640 | 3816816 | 3818006 | norA | Quinolone resistance protein | _ | 2174864, 2173911 |
| BASU_3669 | 3845533 | 3846450 | ydeD | Uncharacterized transporter yded | _ | 11677609, 9311997 |
| BASU_3674 | 3851217 | 3852434 | yfmI | Putative efflux transporter | _ | 15849754, 16850406 |
| BASU_3676 | 3853592 | 3854170 | _ | Putative amino acid transporter (lyse) | _ | _ |
| BASU_3689 | 3863567 | 3864478 | ydfC | Putative drug/metabolite permease | _ | 15849754, 16850406 |
| BASU_3692 | 3867805 | 3869136 | yyaJ | Putative transporter | _ | 15849754, 16850406, 9529885 |
| BASU_3703 | 3877497 | 3878516 | yyaD | Putative integral membrane protein/transporter | _ | 15849754, 16847258, 16850406 |

**Table S5.** List of *plantarum* species-specific genes

| **Label** | **Gene** | **Product** |
| --- | --- | --- |
| BASUv5_0157 | yizA | Hypothetical protein |
| BASUv5_0222 | ybfE | Hypothetical protein |
| BASUv5_0223 | ybfG | Putative pepdidoglycan binding protein |
| BASUv5_0224 | _ | Conserved exported protein of unknown function |
| BASUv5_0257 | ansZ | L-asparaginase 2 (putative lipoprotein) |
| BASUv5_0285 | amyE | Alpha-amylase |
| BASUv5_0414 | mutT | Putative 8-oxo-dGTP diphosphatase |
| BASUv5_0425 | _ | Hypothetical protein |
| BASUv5_0426 | _ | Hypothetical protein |
| BASUv5_0485 | proP | Putative proline/betaine transporter |
| BASUv5_0487 | _ | Hypothetical protein |
| BASUv5_0488 | ydeM | Uncharacterized protein |
| BASUv5_0518 | yrdP | Uncharacterized oxidoreductase |
| BASUv5_0519 | _ | Hypothetical protein |
| BASUv5_0533 | yugM | Uncharacterized membrane protein yugM |
| BASUv5_0563 | yjia | Uncharacterized protein yjiA |
| BASUv5_0717 | yfmK | Putative acetyltransferase |
| BASUv5_0798 | _ | Uncharacterized protein |
| BASUv5_0942 | yhaZ | Putative DNA alkylation repair enzyme |
| BASUv5_1172 | uxaC | Galacturonate isomerase |
| BASUv5_1175 | uxaB | Altronate oxidoreductase |
| BASUv5_1176 | uxaA | Altronate dehydratase |
| BASUv5_1395 | mlnA | MlnA |
| BASUv5_1396 | mlnB | MlnB |
| BASUv5_1397 | mlnC | MlnC |
| BASUv5_1398 | mlnD | MlnD |
| BASUv5_1399 | mlnE | MlnE |
| BASUv5_1400 | mlnF | MlnF |
| BASUv5_1401 | mlnG | MlnG |
| BASUv5_1402 | mlnH | MlnH |
| BASUv5_1403 | mlnI | MlnI |
| BASUv5_1535 | _ | Aminoglycoside phosphotransferase protein |
| BASUv5_1649 | _ | Hypothetical protein |
| BASUv5_1751 | _ | 2-keto-3-deoxygluconokinase / fructokinase |
| BASUv5_1753 | kdgA | KHG/KDPG aldolase |
| BASUv5_1754 | uxuA | D-mannonate dehydratase |
| BASUv5_1755 | uxuB | Fructuronate reductase |
| BASUv5_1756 | endR | Putative HTH-type transcriptional regulator |
| BASUv5_1757 | exuT | Hexuronate transporter |
| BASUv5_1758 | yndG | Conserved hypothetical protein |
| BASUv5_1764 | eglS | Endo-1,4-beta-glucanase |
| BASUv5_1778 | yxjC | Putative permease |
| BASUv5_1854 | _ | Conserved protein of unknown function |
| BASUv5_1953 | ydfR | Putative membrane protein |
| BASUv5_1954 | yrdC | Putative hydrolase |
| BASUv5_2108 | yqjZ | Putative degradation enzyme (oxygenase) |
| BASUv5_2114 | yqjT | Putative lyase |
| BASUv5_2120 | dfnM | DfnM |
| BASUv5_2121 | dfnL | DfnL |
| BASUv5_2122 | dfnK | DfnK |
| BASUv5_2123 | dfnJ | DfnJ |
| BASUv5_2124 | dfnI | DfnI |
| BASUv5_2125 | dfnH | DfnH |
| BASUv5_2126 | dfnG | DfnG |
| BASUv5_2127 | dfnF | DfnF |
| BASUv5_2128 | dfnE | DfnE |
| BASUv5_2129 | dfnD | DfnD |
| BASUv5_2130 | dfnC | DfnC |
| BASUv5_2131 | dfnB | DfnB |
| BASUv5_2132 | dfnX | DfnX |
| BASUv5_2133 | dfnY | DfnY |
| BASUv5_2134 | dfnA | DfnA |
| BASUv5_2135 | _ | Transcription antitermination protein nusG |
| BASUv5_2565 | yjdF | Conserved hypothetical protein |
| BASUv5_2739 | _ | Cupin_1 domain protein (nutrient reservoir activity) |
| BASUv5_2764 | _ | Probable NADPH-dependent FMN reductase |
| BASUv5_2765 | ycaC | Isochorismatase-like hydrolases |
| BASUv5_2920 | _ | LysR family transcriptional regulator |
| BASUv5_2921 | adc | Putative acetoacetate decarboxylase |
| BASUv5_2922 | _ | Putative 2-dehydropantoate 2-reductase |
| BASUv5_3248 | ywqL | Putative deoxyribonuclease V |
| BASUv5_3351 | yitE | UPF0750 membrane protein |
| BASUv5_3369 | _ | Uncharacterized protein |
| BASUv5_3370 | _ | Uncharacterized protein |
| BASUv5_3371 | _ | Uncharacterized protein |
| BASUv5_3372 | _ | Transcription regulator LuxR, C-terminal (gerE-like) |
| BASUv5_3638 | _ | Hypothetical protein |
| BASUv5_3639 | _ | Putative ABC transporter ATP-binding protein |
| BASUv5_3656 | yycA | Uncharacterized protein |
| BASUv5_3683 | _ | Uncharacterized protein |
